# Bioinspired Ether Cyclizations within a π‐Basic Capsule Compared to Autocatalysis on π‐Acidic Surfaces and Pnictogen‐Bonding Catalysts

**DOI:** 10.1002/chem.202101548

**Published:** 2021-07-08

**Authors:** Xiaoyu Hao, Tian‐Ren Li, Hao Chen, Andrea Gini, Xiang Zhang, Stéphane Rosset, Clément Mazet, Konrad Tiefenbacher, Stefan Matile

**Affiliations:** ^1^ Department of Organic Chemistry University of Geneva Quai Ernest Ansermet 30 CH-1121 Geneva Switzerland; ^2^ NCCR Molecular Systems Engineering BPR 1095 Mattenstrasse 24a CH-4058 Basel Switzerland; ^3^ Department of Chemistry University of Basel Mattenstrasse 24a CH-4058 Basel Switzerland; ^4^ Department of Biosystems Science and Engineering ETH Zurich Mattenstrasse 26 CH-4058 Basel Switzerland; ^5^ College of Materials Chemistry and Chemical Engineering Chengdu University of Technology 1 Dongsan Road Erxianqiao Chengdu 610059 P.R. China; ^6^ Shaanxi Key Laboratory of Natural Products and Chemical Biology College of Science Northwest A&F University Xianyang Shi, Yangling 712100 P. R. China

**Keywords:** autocatalysis, anion-π catalysis, capsules, ether cyclization, supramolecular catalysis

## Abstract

While the integration of supramolecular principles in catalysis attracts increasing attention, a direct comparative assessment of the resulting systems catalysts to work out distinct characteristics is often difficult. Herein is reported how the broad responsiveness of ether cyclizations to diverse inputs promises to fill this gap. Cyclizations in the confined, π‐basic and Brønsted acidic interior of supramolecular capsules, for instance, are found to excel with speed (exceeding general Brønsted acid and hydrogen‐bonding catalysts by far) and selective violations of the Baldwin rules (as extreme as the so far unique pnictogen‐bonding catalysts). The complementary cyclization on π‐acidic aromatic surfaces remains unique with regard to autocatalysis, which is shown to be chemo‐ and diastereoselective with regard to product‐like co‐catalysts but, so far, not enantioselective.

## Graphical abstract

**Breaking the rules**: Thanks to their broad responsiveness, epoxide‐opening ether cyclizations are identified as ideal for the direct comparison of supramolecular catalysts. The results suggest that π‐basic supramolecular capsule catalysts are as outstanding as pnictogen‐bonding catalysts in breaking the Baldwin rules with high diastereoselectivity, particularly in cyclization cascades leading to bicyclic products. In clear contrast, chemo‐ and diastereoselective co‐ and autocatalysis is confirmed to be unique for anion‐π interactions.

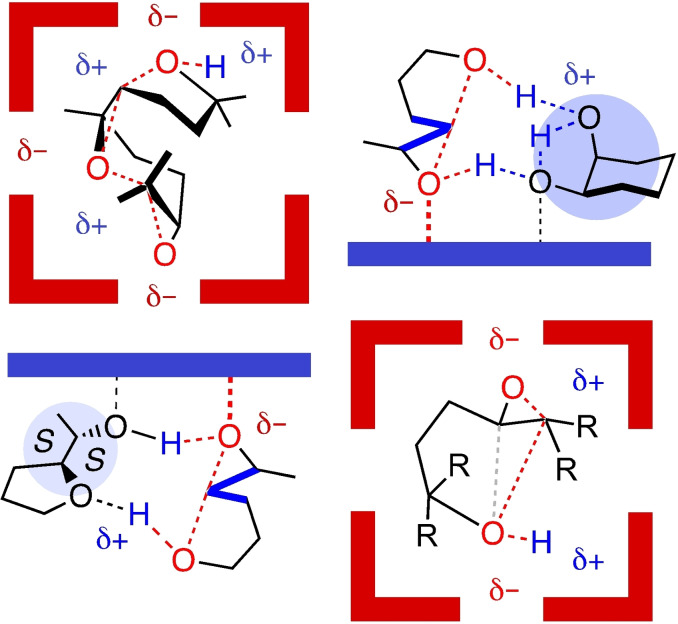

## Introduction

**Figure 1:**
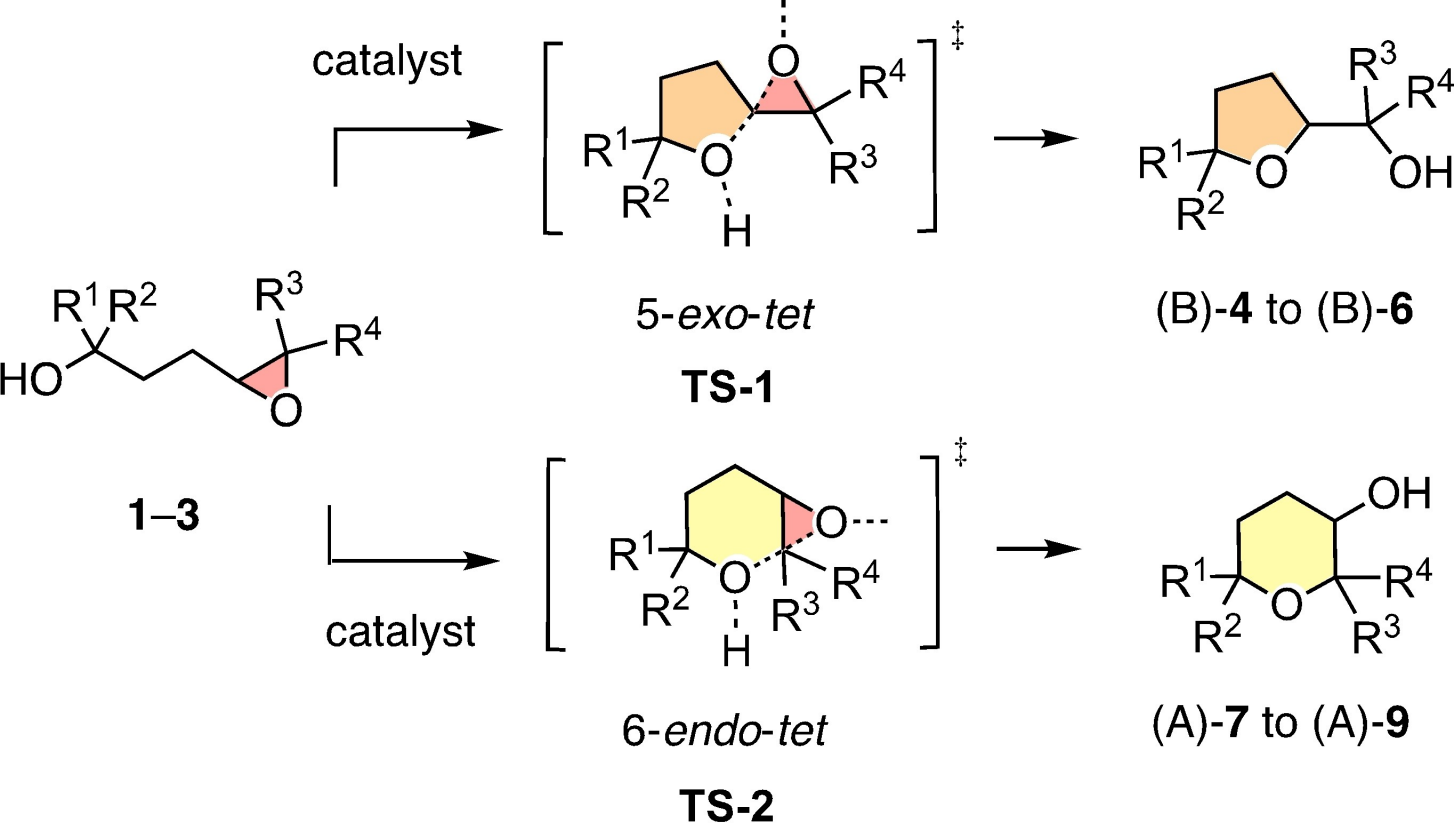
According to the Baldwin rules, ether cyclization of epoxides **1**–**3** through the formal 5‐*exo‐tet* transition state **TS‐1** into (B)‐**4** to (B)‐**6** is favored, whereas the 6‐*endo‐tet* transition state **TS‐2** yields the disfavored anti‐Baldwin (A) products (A)‐**7** to (A)‐**9** (R^1^–R^4^=Me or H, see Figures 3–6, below).

The integration of fundamental, at best new principles from supramolecular chemistry into catalytic systems is an emerging topic of exceptional potential.[^1^, ^2^, ^3^, ^4^, ^5^, ^6^] Offering new ways to get into contact on the molecular level promises access to new reactivity, new structures and new functions, to, hopefully, tackle central challenges that are otherwise intractable. The evaluation of the resulting supramolecular systems catalysts is usually done separately, with different reactions under different conditions. Without contrast from direct comparison with competing approaches, it can thus be difficult to assess the specific added value, the special advantages offered by a given system. In this study, we explore four epoxide‐opening ether cyclizations[^4^, ^7^, ^8^, ^9^, ^10^] for their potential to enable the direct comparison of catalytic systems that have been introduced to operate with general Brønsted acids and hydrogen bonds,[^11^, ^12^, ^13^] general Lewis acids and pnictogen bonds,[^4^, ^14^, ^15^, ^16^] cation‐π[^2^, ^6^, ^12^] and anion–π interactions,[^3^, ^17^] and encapsulation,[^2^, ^6^, ^16^, ^18^, ^19^] co‐catalysis and autocatalysis[^19^, ^20^, ^21^] (Figure 1). Epoxide‐opening ether cyclizations are of interest for this purpose because they offer maximal responsiveness. Possibilities cover activation of nucleophile, electrophile and leaving group, shifts from more S_N_2‐ to more S_N_1‐type mechanisms, intriguing chemoselectivity centered around the Baldwin rules,^[22]^ and diverse stereoselectivity, from *cis*‐*trans* diastereomers, including ring fusion, to enantioselectivity.^[4]^

Epoxide‐opening ether cyclizations are classics in organic chemistry.[^4^, ^7^, ^8^, ^9^, ^10^] This interest originates from polyether natural products. These most complex privileged scaffolds are expected to originate from cascade cyclizations of highest sophistication. According to the Baldwin rules, 4,5‐epoxy alcohols such as **1**–**3** prefer to cyclize through 5‐*exo‐tet* transition states like **TS‐1** into THFs or oxolanes such as **4**–**6**, while 6‐*endo‐tet* cyclizations through **TS‐2** into THPs or oxanes **7**–**9** are disfavored (Figure 1). Similar *exo* selectivity applies for essentially all ring sizes. In biosynthesis, cascades cyclizations that follow the Baldwin rules occur for instance in monensin A, an oligo‐THF ion carrier.^[10]^ Most charismatic is the cascade cyclization of eleven rings with anti‐Baldwin selectivity in every step that has been proposed early on by Nakanishi to lead to brevetoxin B.^[7]^ Bioinspired polyether cascade cyclizations on the one hand and strategies to violate the Baldwin rules have been explored extensively in many groups.[^8^, ^9^, ^10^] Most efforts have focused on specific modifications of substrate structures, while the promise of high responsiveness in chemo‐ and stereoselectivity to probe for advantages of new catalysts has received less attention. Pioneering examples include cavitands^[8]^ and catalytic antibodies^[9]^ and for Baldwin and anti‐Baldwin cyclization of mono‐epoxides, respectively.

**Figure 2:**
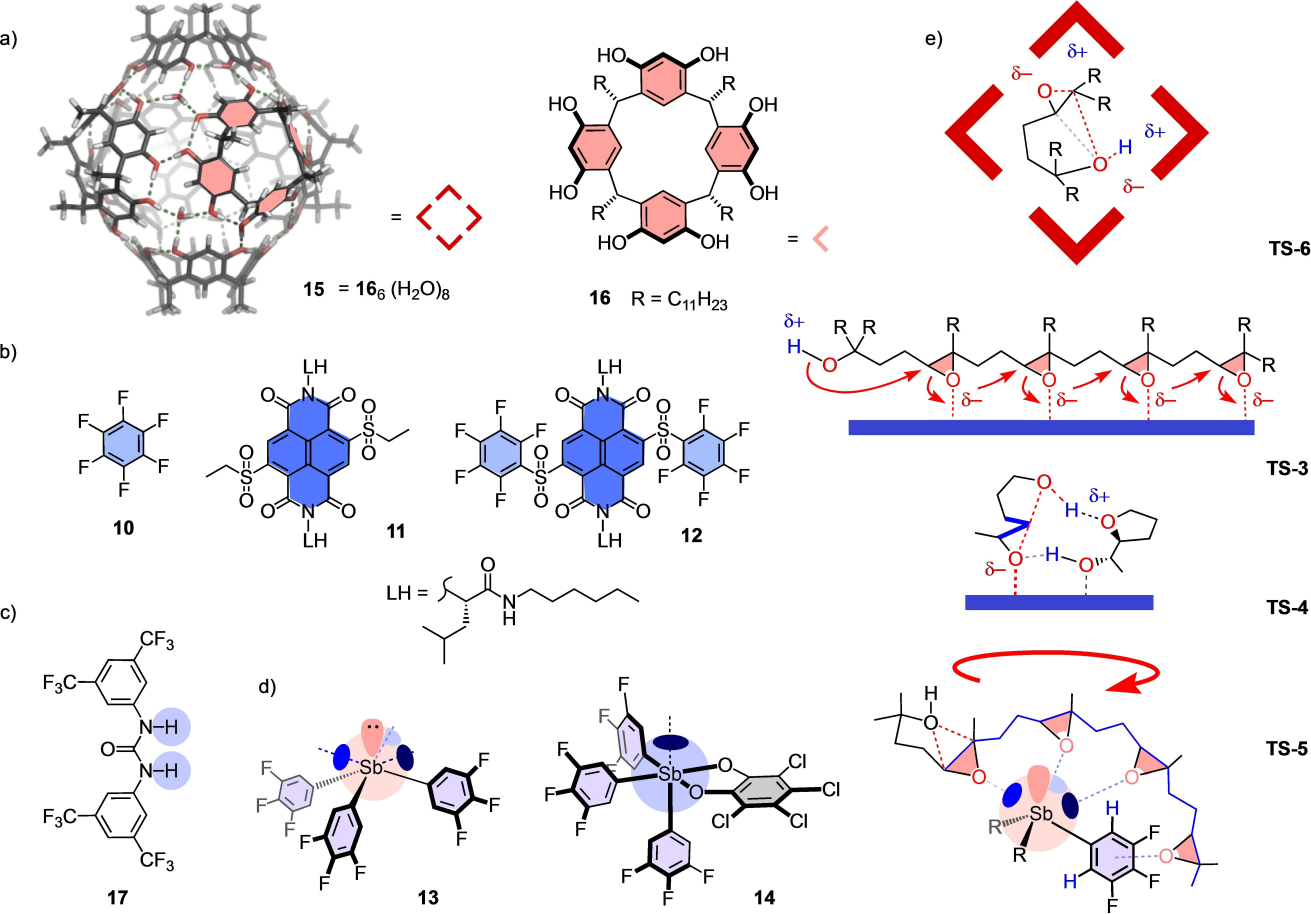
a)–d) Structures of catalysts **10–17**, with e) tentative transition states outlining conceivable characteristics, including cation–π interactions in confined space of π‐basic capsules **15** (**TS‐6**), anion–π interactions, cascade cyclization of a tetraepoxide^[24]^ (**TS‐3**) and autocatalysis (**TS‐4**) on π‐acidic surfaces of **10**–**12**, and multiple pnictogen bonding for cascade cyclization of a tetraepoxide^[4]^ on antimony(III) catalyst **13** (**TS‐5**).

More recently, anion‐π catalysis of polyether cascade cyclizations has been explored as possible counterpart of steroid cyclization as most spectacular expression of cation‐π biocatalysis.[^23^, ^24^] Anion‐π catalysis has been introduced in 2013 as the unorthodox counterpart of cation‐π catalysis to stabilize anionic rather than cationic transition states on π‐acidic rather than π‐basic surfaces.^[3]^ The delocalized nature of anion‐π interactions could be expected to serve best in stabilizing coupled transition states that involve charge displacements over large distances. Up to four epoxides have been cyclized on π‐acidic surfaces (Figure 2b).^[24]^ As outlined in **TS‐3**, anion‐π catalysis is expected to activate the epoxide leaving group by stabilizing the released alcoholate anion. Cascade cyclizations on π‐acidic surfaces of simple primary anion–π catalysts **10**–**12** were mostly Baldwin selective and showed autocatalytic behavior. As outlined in **TS‐4**, computational simulations suggest that autocatalysis originates from two hydrogen bonds between transition state and product that activate the nucleophile and the leaving group, respectively.^[23]^

Pnictogen‐bonding catalysis has been introduced in 2018 as noncovalent counterpart of Lewis acid catalysis,[^4^, ^14^] analogous to hydrogen‐bonding catalysis as non‐covalent counterpart of Brønsted acid catalysis.[^11^, ^12^, ^13^] Probed with polyether cascade reactions, pnictogen‐bonding catalysts operating on the Sb^III^ level like **13** and on the Sb^V^ level like **14** were not autocatalytic but excelled with efficient violation of the Baldwin rules (Figure 2d). Sb^III^ catalysts appeared particularly attractive because three σ holes around a central lone pair could be involved in catalyzing the cascade cyclization, as outlined in **TS‐5**.

Anti‐Baldwin selectivity and autocatalysis identified as signature properties in pnictogen‐bonding and anion‐π catalysis, respectively, called for comparison with other motifs in systems catalysis. Key questions particularly with regard to anion‐π autocatalysis concerned uniqueness, chemoselectivity (i. e., self‐replication^[20]^), and stereoselectivity (i. e., asymmetric autocatalysis^[21]^). In the following, these questions are addressed focusing on four complementary cyclizations catalyzed within molecular capsule **15**.[^2^, ^25^] Capsule **15** self‐assembles from six resorcin[4]arenes **16** and eight molecules of water (Figure 2a).^[26]^ Proton transfer from these water molecules can trigger acid catalysis within the confined interior that can be supported by cation‐π interactions of the resulting intermediates with the inner surface of the π‐basic capsule.^[27]^ The cyclization of mono‐ and sesquiterpenes with capsule **15** has provided access to intriguing chemoselectivity.^[2]^ In the following, we show that epoxide‐opening ether cyclizations within the confined, Brønsted acidic and π‐basic interior of capsule **15** is possible and characterized by fast conversion and globular transition states like **TS‐6** in response to spatial confinement. The results are anti‐Baldwin chemoselectivity that rivals the so far unique pnictogen‐bonding catalysts and largely exceeds hydrogen‐bonding control **17**. In clear contrast, autocatalysis remains unique for π‐acidic surfaces and is shown to be chemo‐, diastereo‐ but not enantioselective.

## Results and Discussion

**Figure 3:**
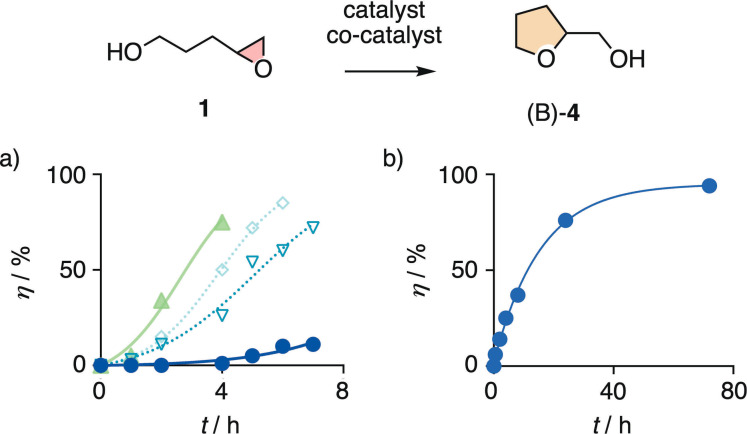
Reaction kinetics at room temperature for the conversion of **1** (1 M) into (B)‐**4** in the presence of a) anion–π catalyst **10** (solvent) and 0 (•), 0.5 (▿), 1.0 (◊), and 2.0 (▴) equiv. of **4** added at the beginning of the reaction (autocatalysis curve fit), and b) capsule **15** (first‐order curve fit; (A)‐**7** not observed, Figure 1).

The cyclization of 4,5‐epoxy alcohol **1** afforded the Baldwin‐compatible *5‐exo‐tet* oxolane product (B)‐**4** under all tested conditions (Figure 3 and Figures S1–S5 in the Supporting Information). Within capsule **15**, the cyclization was comparably slow and did not involve autocatalysis (Figures 3b and S5). A *t*_50_=10.8±0.8 h was obtained (Table S1). Without additional methyl groups, the nucleophile in **1** is comparably strong. Catalysis will thus be mostly needed to activate electrophile and leaving group, which, as expected, is less efficient within the π–basic capsule.

In hexafluorobenzene **10** as a solvent catalyst, the conversion was even slower, reaching around 10 % in 7 h (Figure 3a, •, and Figure S1). Fitting of the sigmoidal curve for product formation with time to autocatalysis gave a rate enhancement of *k*_autocat_/*k*_cat_=190±55 M^−1^, and an extrapolated *t*_50_ ≫10 h. In the presence of increasing concentrations of product **4** from the beginning, the initial rate of cyclization accelerated up to *k*_cat_/*k*_cat(0)_=30±10 (Figures 3a, S2–S4, Table S1). All kinetics profiles in **10** were sigmoidal, clearly different from the apparent first‐order profile in capsule **15**. Autocatalysis rate enhancement decreased down to *k*_autocat_/*k*_cat_=15±5 M^−1^ in the presence with 2.0 equivalents of product **4** due to the faster conversion from the beginning (Figure 3a, ▴). Half‐life times decreased with increasing product added at the beginning. The *t*_50_=2.8±0.1 h reached in **10** with 2.0 equivalents was almost 4 times shorter than the *t*_50_=10.8±0.8 h in capsule **15** (Table S1). These trends indicated that the activation of epoxide opening by alcoholate‐π interactions with the weakly π acidic **10** alone is insufficient to cause significant rate enhancements, while further leaving group activation with a hydrogen bond to the product on the π surface affords significant anion‐π autocatalysis. This interpretation was consistent with the computational insights summarized in **TS‐4** (Figure 2e). In the absence of π‐acidic aromatic surfaces, product **4** did not catalyze the conversion of substrate **1**.

**Figure 4:**
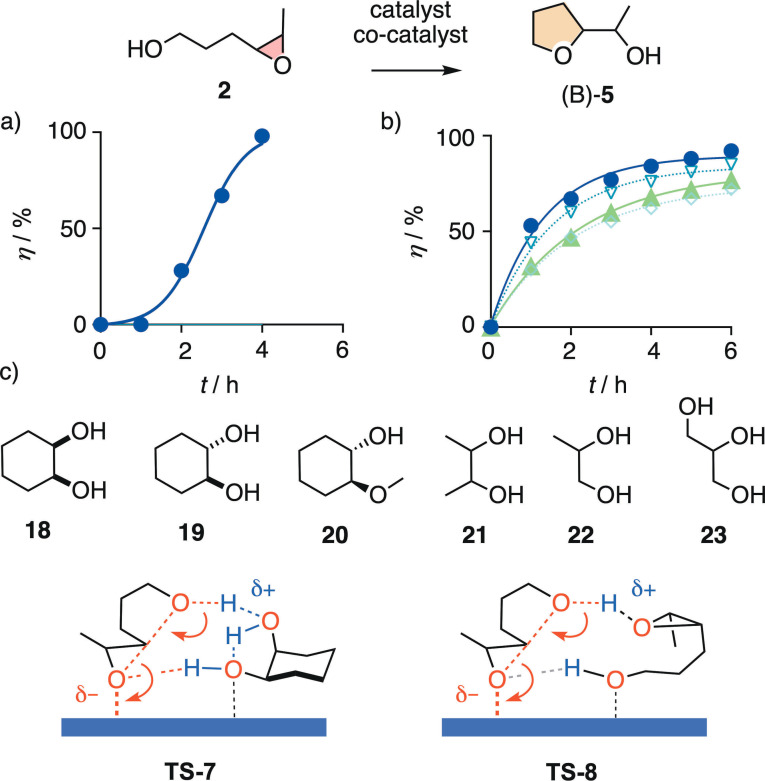
Reaction kinetics at room temperature for the conversion of **2** (1 M) into (B)‐**5** in the presence of a) anion–π catalyst **10** (solvent; autocatalysis curve fit) and b) capsule **15** and 0 (•), 0.25 (▿), 0.5 (◊), and 1.0 (▴) equiv. of **5** added at the beginning of the reaction (first‐order curve fit; (A)‐**8** not observed). c) Product mimics **18**–**23** tested as co‐catalysts of NDI **11** (Table 1), with notional transition states **TS‐7** and **TS‐8** for **18** and substrate **2** as co‐catalysts for anion–π catalysts, respectively.

Like substrate **1**, the cyclization of the 4,5‐epoxy alcohol **2** with an extra methyl in *cis* position on the epoxide gave the Baldwin product (B)‐**5** exclusively (Figures 4 and S6–S10). In capsule **15**, the reaction was clearly faster (Figure 4b vs. 3b). The *t*_50_=10.8±0.8 h for **1** dropped to *t*_50_=0.9±0.1 h for **2** (Table S2). Substrate decay was first‐order‐like rather than sigmoidal. The addition of extra product at the beginning slowed down rather than accelerated the reaction, which was consistent with mildly competitive product encapsulation (Figures 4b, 2e). Substrate lifetimes increased up to *t*_50_=1.6±0.1 h in the presence of one equivalent of product **5** (Table S2).

Conversion of **2** in hexafluorobenzene **10** was also much faster than **1** and strongly autocatalytic (Figure 4a). The *t*_50_=2.6±0.1 h for **2** on the π surface was longer than *t*_50_=0.9±0.1 h in the capsule (Table S2). The characteristic rate enhancement by autocatalysis calculated to *k*_autocat_/*k*_cat_=135±20 M^−1^, which was slightly below the *k*_autocat_/*k*_cat_=190±55 M^−1^ obtained for **1**. Unlike **4** added to **1**, the addition of product **5** at the beginning of the cyclization of **2** in **10** did not accelerate conversion. Poorly reproducible decreases were observed instead, suggesting that the formed complexes aggregated and possibly precipitated. This behavior is neither surprising nor novel. Autocatalysis on π‐acidic surfaces in general strongly depends on substrate and anion–π catalyst, with several parameters including solubility and concentration (and minor product impurities in the substrate) contributing to the final outcome.

Replacement of the weak anion–π solvent catalyst **10** by the much stronger anion‐π catalyst **11** has been shown to afford impressive autocatalysis with **2**, characterized by excellent responsiveness to the addition of product at the beginning of the reaction.^[23]^ This combination thus provided a convenient minimalist system to elaborate on the selectivity of primary anion‐π autocatalysis with regard to the structure of the product. This question was important to determine whether or not the catalytic system could be considered as self‐replicating.^[20]^ The computational model **TS‐4** of anion‐π autocatalysis^[23]^ implies that one hydrogen‐bond acceptor and one hydrogen‐bond donor are needed to activate nucleophile and leaving group, respectively (Figure 2e).

In this context, product mimics **18**–**23** were tested as possible co‐catalysts. In each experiment, 100 mol% were added at the beginning of the conversion of substrate **2** on 10 mol% anion‐π catalyst **11**, and substrate consumption after five days was compared to the 70 % obtained without co‐catalyst (Table 1). Most additives decreased rather than increased conversion, down to 39 %, that is almost half, in the presence of the vicinal diol **21** (entry 8). Full conversion was observed only for **20**, featuring vicinal hydroxy and methoxy groups in rigidified *trans* position (entry 7) to activate nucleophile and electrophile, respectively, by hydrogen bonding on the π‐acidic surface.

**Table 1:** Co‐catalyst screening for substrate **2**.^[a]^

Entry	Co−C^[b]^	mol %^[c]^	C^[d]^	*c*_S_ [M]^[e]^	*η* [%]^[f]^
1	–	–	**11**	0.84	70
2	CD_3_OD	100	**11**	0.84	50
3	Glycol	100	**11**	0.84	48
4	IPA^[g]^	100	**11**	0.84	48
5	**18**	100	**11**	0.46	89
6	**19**	100	**11**	0.46	42
7	**20**	100	**11**	0.46	100
8	**21**	100	**11**	0.84	39
9	**22**	100	**11**	0.84	56
10	**23**	100	**11**	0.84	40

[a] Conditions: Substrate **2**, concentration *c*_S_ as indicated, 10 mol% catalyst **11**, 100 mol% co‐catalyst, CD_2_Cl_2_, 20 °C, 5 d. [b] Co‐catalysts, Figure 4c. [c] Concentration co‐catalyst, in mol % relative to substrate concentration. [d] Catalyst (Figure 2). [e] Substrate concentration. [f] Substrate conversion [%] from ^1^H NMR spectra of product mixtures. [g] Isopropyl alcohol.

Replacement of the methoxy acceptor in **20** by a hydroxy donor in *trans* 1,2‐cyclohexanediol **19** converted the co‐catalyst into a respectable inhibitor. Conversion was with 42 % about as poor as the 39 % record of the more flexible vicinal diol **21** (entries 6, 9). In the context of the computational transition‐state model, this inversion of activity could indicate that the new hydroxy group acts as hydrogen bond donor rather than acceptor to inactivate rather than activate the nucleophile in the substrate. Inversion of the *trans* configuration in diol **21** to *cis* configuration in **18** restored co‐catalyst activity up to 89 % conversion (entry 5). This significant diastereoselectivity suggested that in *cis* **18**, one alcohol acts as hydrogen‐bond acceptor to activate the nucleophile and as intramolecular hydrogen‐bond donor to strengthen the vicinal alcohol as activator of the epoxide leaving group, as outlined in **TS‐7** (Figure 4c). This intramolecular hydrogen‐bond could further serve as a relay to facilitate the proton transfer from nucleophile to leaving group needed to finalize the reaction. Intramolecular hydrogen bonds in *cis* 1,2‐cyclohexanediol **18** have been reported to be shorter (2.26 Å) than in the *trans* isomer **21** (2.33 Å), and hydrogen‐bonded cyclic dimers analog to **TS‐7** have been confirmed to exist in solution, in equilibrium with monomers and higher oligomers.^[28]^

The non‐linear dependence of conversion rates on substrate concentration has already been shown to identify substrate **2** as co‐catalyst of its own conversion, as outlined in **TS‐8**.^[23]^ The very existence of autocatalysis, however, demonstrates that substrates, in general, are weaker co‐catalysts than products for anion‐π catalysis of epoxide‐opening ether cyclizations. Without anion‐π catalyst, none of the identified co‐catalysts, including substrate and product, catalyzed the cyclization significantly. Overall, these findings thus suggested that the autocatalysis of ether cyclization on π‐acidic surfaces does not strictly fulfill the selectivity expected for self‐replication,^[20]^ but that the selectivity restrictions for co‐catalysis are nevertheless remarkably high.

**Figure 5:**
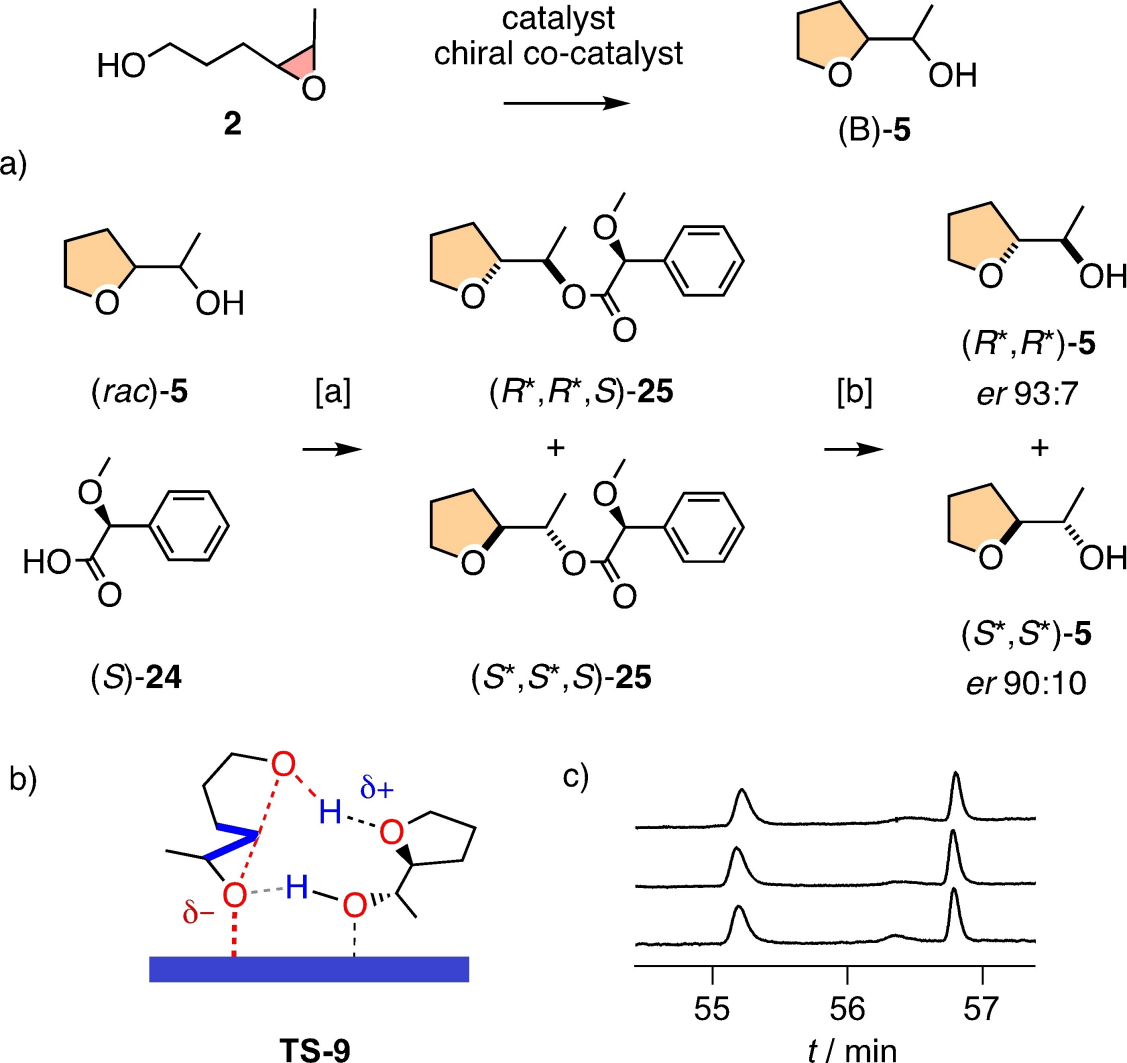
a) Synthesis of enantioenriched products as chiral co‐catalysts for the conversion of **2** (1.0 M) into (B)‐**5** in the presence of anion‐π catalyst **11** (10 mol%, CD_2_Cl_2_, RT), with b) notional **TS‐9** indicating the position of stereogenic centers and c) chiral GC profiles for the consumption of enantiomers of substrate **2** after 25, 45, and 73 h (top to bottom) in the presence of anion‐π catalyst **11** and 0.5 equiv. (S*,S*)‐**5**. [a] EDC, DMAP, CH_2_Cl_2_, 30+37 %. [b] K_2_CO_3_, MeOH, 30 and 20 %.

The same minimalist system was attractive to probe for stereoselectivity, that is asymmetric autocatalysis^[21]^ (Figure 5). Enantioenriched product **5** was synthesized by esterifying the racemic alcohol (*rac*)‐**5** with enantiopure acid (*S*)‐**24** (Figure 5a). The resulting diastereomers **25** were separated and hydrolyzed to yield the enantiomers (R*,R*)‐**5** and (S*,S*)‐**5** with, according to chiral GC analysis, *er*=93 : 7 and *er*=90 : 10, respectively. These enantioenriched products of unknown absolute configuration were then used as co‐catalysts for the conversion of racemic substrate **2** with anion–π catalyst **11** at concentrations of 0.5 and 1.0 equivalents. The consumption of the substrate enantiomers was followed over time by chiral GC. Significant differences between the consumption of the two enantiomers in the presence of enantioenriched products could not be observed at any time in the reaction (Figure 5c). Inspection of the computational model of the transition state, that is, **TS‐9**, suggested that the stereogenic centers in product **5** are presumably too far from substrate **2** to induce asymmetric autocatalysis (Figure 5b).

**Figure 6:**
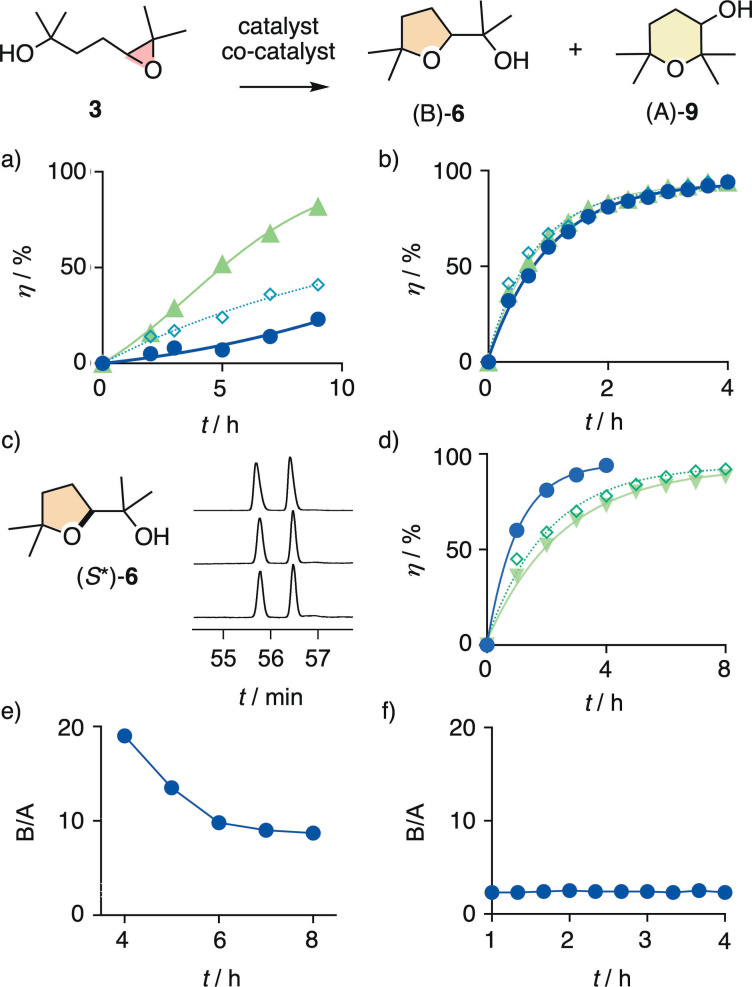
a) Reaction kinetics for the conversion of **3** (1 M) into (B)‐**6** and (A)‐**9** in the presence of a) anion–π catalyst **10** as the solvent and 0 (•), 0.1 (◊) and 1.0 (▴) equiv. of (*rac*)‐**6** added at the beginning of the reaction (RT, autocatalysis curve fit). b) Same with capsule **15** (10 mol% in CDCl_3_) and 0 (•), 0.25 (◊) and 1.0 (▴) equiv. of (*rac*)‐**6** or d) (*rac*)‐**9** (first‐order curve fit). c) Chiral GC profiles for the consumption of enantiomers of substrate **3** after 2 (28 %), 3 (51 %), and 5 h (87 %; top to bottom) in the presence of anion–π catalyst **10** (solvent) and 10 equiv. of (S*)‐**6**. e) B/A ratio of products (B)‐**6** and (A)‐**9** during the conversion of **3** in the presence of anion–π catalyst **10** and f) capsule **15**.

Unlike substrates **1** and **2**, the cyclization of the 4,5‐epoxy alcohol **3** with four extra methyl groups around nucleophile and electrophile gave the Baldwin oxolane product (B)‐**6** together with the anti‐Baldwin oxane product (A)‐**9**, in B/A ratios that varied from catalyst to catalyst (Figure 6). This emergence of anti‐Baldwin selectivity would be consistent with an increasingly S_N_1‐like mechanism with a tertiary carbocation‐like intermediate. In capsule **15**, the reaction further accelerated from *t*_50_=10.8±0.8 h for **1** and *t*_50_=0.9±0.1 h for **2** to *t*_50_=0.70±0.03 h for **3** (Figure 6b, Table S3), possibly due to stabilization by cation–π interactions within the capsule (**TS‐6**, Figure 2e). Reaction kinetics remained first order without any indication of autocatalysis.

Conversion of **3** in hexafluorobenzene **10** was about as slow as for **1**, reaching 20 % conversion in 9 h (Figures 6a, S11). The rate enhancement by autocatalysis calculated to *k*_autocat_/*k*_cat_=11±6 M^−1^ (Table S3). The addition of product **6** at the beginning of the cyclization of **3** in **10** accelerated the reaction significantly. Exactly as for **1**, the kinetics profiles in **10** were all sigmoidal and differed clearly from the exponential substrate decay in capsule **15** (Figures 6a and S11–S13). The autocatalysis rate enhancements decreased to *k*_autocat_/*k*_cat_=5.0±0.5 M^−1^ with 1.0 equivalents because of increasing initial rates (Figure 6a, ▴). As a consequence, half‐live times of **3** steadily decreased, from *t*_50_=≫10 h without to *t*_50_=4.6±0.2 h with 1.0 equivalent of product **6** added at the beginning. This product‐assisted performance of the weak anion‐π solvent catalyst **10** was still inferior to the *t*_50_=0.70±0.03 h within capsule **15** (Table S3).

Asymmetric autocatalysis was tested as described for **2**. However, the presence of 0.5 to 10 equivalents enantioenriched product (S*)‐**6** did, according to chiral GC analysis, not result in enantioselective consumption of substrate **3**, also when measured at 0 °C (Figure 6c). As with the stronger anion‐π catalyst **11** for substrate **2**, the weaker anion‐π solvent catalyst **10** thus failed to mediate asymmetric autocatalysis for **3** in the presence of enantioenriched product (S*)‐**6**.

General Brønsted and Lewis acid catalysis of the cyclization of **3** followed the Baldwin rules with reasonably high fidelity. The B/A ratio of Baldwin oxolane **6** and anti‐Baldwin oxane **9** was reliably around 9 : 1 (Table 2, entries 1–3).^[4]^ Anion–π catalysts **10** and **12** did not change this intrinsic selectivity (entries 4, 5, Figures S11–S13).[^23^, ^24^] Anion–π catalyst **12** was much faster than general Brønsted and much slower than general Lewis acid catalysts.^[4]^ In clear contrast, capsule **15** more significantly violated the Baldwin rules (entry 7, Figures S14–S18). The B/A=2 : 1 approached violations of the Baldwin rules observed previously^[4]^ only for pnictogen‐bonding catalysts **13** and, most impressively, **14** (entries 8, 9). A more developed hydrogen‐bonding catalyst **17** ^[13]^ was tested as a negative control and confirmed to perform in the standard B/A ∼9:1 region (entry 6). Intriguingly, Rebek and coworkers studied the same reaction long ago within cavitands equipped with an inward acid catalyst.^[8]^ Only Baldwin product **6** was observed, anti‐Baldwin oxane **9** and autocatalysis were not mentioned.

**Table 2:** Catalyst comparison on the mono‐epoxide level, that is, substrate **3**.^[a]^

Entry	C^[b]^	*c* [mol %]^[c]^	*T* [°C]^[d]^	*t* ^[e]^	*η* [%]^[f]^	B/A (**6**/**9**)^[g]^
1	AcOH	100	RT	18 h	100	92 : 8
2	SbCl_3_	1	RT	<5 min	100	87 : 13
3	BF_3_OEt_2_	10	RT	<5 min	100	84 : 16
4	**10**	867^[h]^	RT	9 h	20	90 : 10
5	**12**	5	RT	30 h	100	89 : 11
6	**17**	20	40	6 d	63	88 : 12
7	**15**	10	RT	4 h	100	66 : 34
8	**13**	100	40	24 h	81	60 : 40
9	**14**	1	RT	<5 min	100	30 : 70

[a] Data except for entries 3, 6 and 7 from references.[^4^, ^23^, ^24^] [b] Catalysts (Figure 2). [c] Concentration catalyst, in mol % relative to concentration of substrate **3** (1.0 M in CD_2_Cl_2_). [d] Reaction temperature; RT: room temperature. [e] Reaction time. [f] Substrate conversion, in percent, from ^1^H NMR spectra of product mixtures. [g] Ratio of yield of Baldwin (B) product **6** divided by yield of anti‐Baldwin (A) product **9**, from ^1^H NMR spectra of product mixtures. [h] Solvent catalysis.

Beyond a substantial violation of the Baldwin rules, capsule **15** was further remarkable with regard to catalytic efficiency. The supramolecular catalyst was much faster than the hydrogen‐bonding control **17**, general Brønsted acid catalysis, pnictogen‐bonding catalyst **13** and all anion‐π catalysts (entries 1, 4–8). Only general Lewis acid catalysis and the hypervalent Sb^V^ pnictogen‐bonding catalyst **14** were naturally faster (entries 2, 3, 9).

The addition of the Baldwin product **6** at the beginning of the reaction caused a negligibly small acceleration of the cyclization of **3** within capsule **15**, and the apparent first‐order kinetics profile did not change (Figures 6b and S14–S16). This was in clear contrast to the dramatic rate enhancements caused by products added to anion–π catalyst **10** (see above, Figure 6a). This clear difference confirmed that autocatalysis is a unique characteristic of anion‐π catalysis and does not occur within capsule **15**. However, the addition of the anti‐Baldwin product **9** at the beginning of the reaction slowed down the reaction more substantially, with the half‐life time raising up to *t*_50_=1.9±0.2 h with 1.0 equivalent of anti‐Baldwin **9** (Figures 6d, S14, S17, and S18). This result suggested that the more globular anti‐Baldwin product **9** binds better within capsule **15** than the Baldwin product **6**. This selective product recognition was in nice agreement with the comparably good B/A=2 : 1 obtained for cyclization of **3** within capsule **15**. The notional **TS‐6** qualitatively summarizes these conclusions and indicates possible interactions with the π‐basic arenes and the acidic water of the capsule (Figure 2e).

The B/A=2 : 1 for the cyclization of **3** within capsule **15** did not change during the course of the reaction (Figure 6f). With anion‐π catalysts **10**, the B/A ratio decreased from a very high B/A ∼20 at the beginning of the reaction toward the final B/A=9 : 1 (Figure 6e). With anion–π catalysis, on the one hand, this decreasing B/A ratio supported direct contact of the product with substrate and catalyst, as already demonstrated by rate enhancements upon product addition (Figure 6a). The question whether the substrate co‐catalyst increases Baldwin selectivity or the product co‐catalyst increases anti‐Baldwin selectivity is interesting but cannot be answered at this point, also because the changes are comparably small and Baldwin selectivity overall high. The constant B/A values during cyclization of **3** within capsule **15**, on the other hand, did not support direct contact between product, substrate and catalyst. This conclusion confirmed that the deceleration caused by anti‐Baldwin products originates from competitive inhibition, that is encapsulation of product **9** rather than substrate **3** within capsule **15**. However, substrate and particularly transition‐state encapsulation are overall favored over product encapsulation, otherwise product inhibition would be observed.

**Figure 7:**
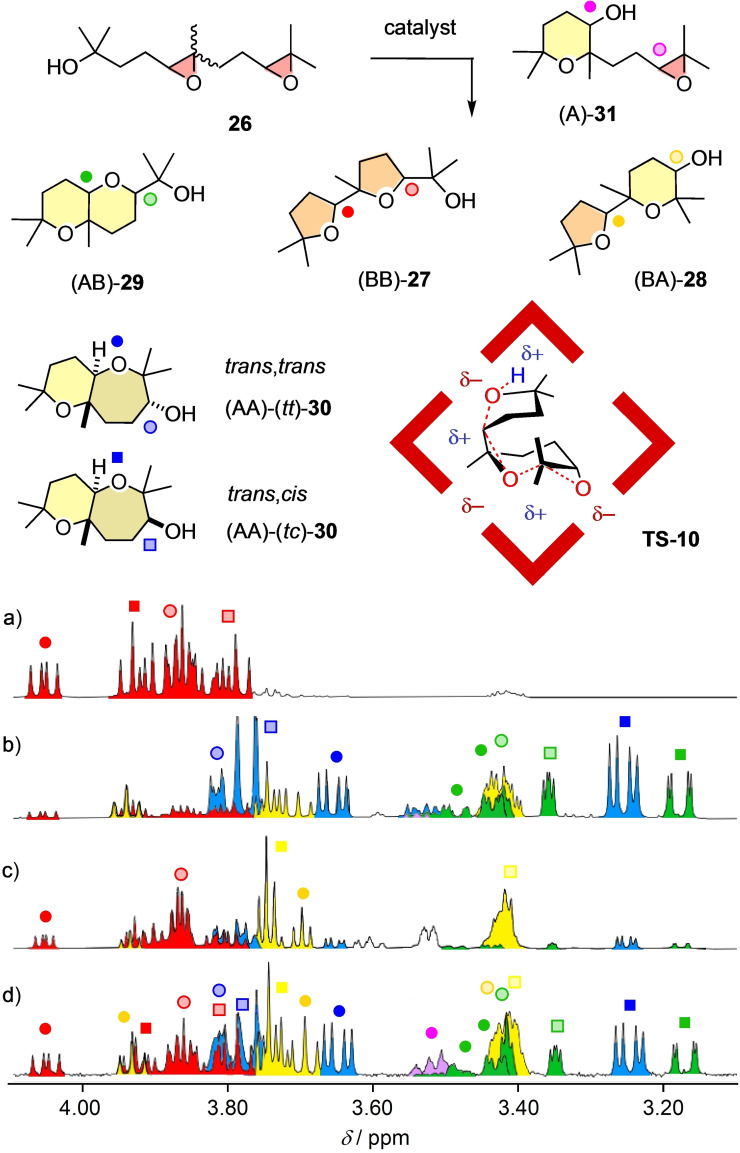
Signature region of the ^1^H NMR spectra of the reaction mixtures obtained from **26** in the presence of a) AcOH (100 mol%, 40 °C, 18 h),^[4]^ b) Sb^V^ catalyst **14** (1 mol%, RT, 2 h),^[4]^ c) capsule **15** (10 mol%, 30 °C, 4 d), and d) Sb^III^ catalyst **13** (500 mol%, 60 °C, 2d),^[4]^ with assignments to products **27**–**31** and a tentative **TS‐10** to rationalize an apparent selectivity for (BA)‐**28** within capsule **15**.

Diepoxide **26** was selected as fourth and final substrate of this comparative study because the B/A chemoselectivity is richer than with monomers but still understandable in NMR spectra of product mixtures (Figure 7).^[4]^ The diepoxide substrate **26** was usually used as mixture of *cis*/*trans* isomers with the 6 : 4 ratio of the commercially available synthetic precursor. The product mixtures can contain the oxolane dimer (BB)‐**27** resulting from a cascade cyclization following the Baldwin rules, the oxolane‐oxane dimer (BA)‐**28** resulting from a Baldwin cyclization followed by an anti‐Baldwin cyclization, and the fused bicycles (AB)‐**29** and (AA)‐**30** produced by an anti‐Baldwin cyclization followed by a Baldwin and an anti‐Baldwin cyclization, respectively. The presence of the intermediate (A)‐**31** is indicative for the directionality of the cascade cyclization from the nucleophile toward the electrophiles. The ^1^H NMR spectra are further complicated by the presence of many diastereomers. For (AA)‐**30**, two *trans*‐fused isomers have been isolated and identified as the isomers (AA)‐(*tt*)‐**30** and (AA)‐(*tc*)‐**30** with methyl and hydroxyl in *trans* and *cis* relation relative to the oxepane ring, respectively.^[4]^

In the ^1^H NMR spectrum of product mixtures, the signals from different products are best separated in the region from 4.1 to 3.1 ppm.^[4]^ This region was thus considered as fingerprint region for the characterization of new catalysts. The ^1^H NMR spectrum of the assigned mixture obtained from **26** with Sb^III^ catalyst **14** was taken as general reference (Figure 7d).^[4]^ Catalysis of the minimalist cascade cyclization of epoxide **26** with general Brønsted and Lewis acids yielded almost only Baldwin products (BB)‐**27** without any contributions from autocatalysis (Figure 7a, Table 3, entries 1, 2, BB/BA=97 : 3 for AcOH).^[24]^ Also anion–π catalyst **12** showed only little deviation from the Baldwin rules (entry 3, BB/BA=87 : 13, from *cis‐* **26**) but unusually strong autocatalysis (*k*_autocat_/*k*_cat_=3.0×10^4^ M^−1^).^[24]^ Failing to mediate autocatalysis, pnictogen‐bonding catalysts **13** and **14** have so far been unique in violating the Baldwin rules substantially.^[4]^ Significant amounts of (AB)‐**29** and (AA)‐**30** were obtained with both, Sb^V^ **14** producing most (AA)‐(*tc*)‐**30** (Figure 7b), Sb^III^ **13** more (AA)‐(*tt*)‐**30** (Figure 7d).^[4]^

**Table 3:** Catalyst comparison on the diepoxide level, that is, substrate **26**.^[a]^

Entry	C^[b]^	*c* [mol %]^[c]^	*T* [°C]^[d]^	*t* ^[e]^	*η* [%]^[f]^	P^[g]^
1	AcOH	100	40	18 h	100	BB (**27**)^[h]^
2	SbCl_3_	100	RT	<5 min	100	BB (**27**)
3	**12**	20	RT	20 h	100	BB (**27**)^[i]^
4	**15**	10	30	4 d	>80	BA (**28**)^[j]^
5	**13**	500	60	2 d	88	AA (**30**), (AB, **29**)
6	**14**	1	RT	2 h	100	AA (**30**), (AB, **29**)

[a] Data for entries 1–3, 5, 6 from references.[^4^, ^24^] [b] Catalysts (Figure 2). [c] Concentration catalyst, in mol % relative to concentration of substrate **26** (250 mM in CD_2_Cl_2_). [d] Reaction temperature; RT: room temperature. [e] Reaction time. [f] Substrate conversion, in percent, from ^1^H NMR spectra of product mixtures. [g] Selectivity, indicating main product P and, in parenthesis, important side products, Figure 7. [h] BB/BA=97 : 3. [i] *cis* epoxide substrate, BB/BA=87 : 13. [j] Obtained with high diastereoselectivity.

Conversion of **26** within capsule **15** afforded (BA)‐**28** as the main product with high apparent diastereoselectivity (Figures 7c, S19). Integration of the two separated peaks at 3.76 ppm and 3.70 ppm suggested a *dr* ∼75 : 25 for the two diastereomers of (BA)‐**28**. According to the fingerprint region on the ^1^H NMR spectrum, only the one (BB)‐**27** diastereomer with distinct signals above 4.00 ppm appeared as a minor side product. Comparison of the total integration for (BA)‐**28** diastereomers with the (BB)‐**27** diastereomer at 4.09 ppm gave an apparent BB:BA=15 : 85. This represents a so far unique inversion of selectivity compared to BB/BA=97 : 3 for AcOH^[24]^ or BB/BA=87 : 13 for anion–π catalyst **12**. The formation of (AB)‐**29** and (AA)‐**30**, both produced from an initial anti‐Baldwin cyclization, is almost completely suppressed. Comparison of the total integration for (BA)‐**28** diastereomers compared to integration for all other regioisomers suggested that within capsule **15**, the (BA)‐**28** diastereomers are obtained from **26** (*cis*/*trans* ∼6 : 4) in 69 % conversion yield, with the major diastereomer being obtained in 50 %. This diastereoselective formation of isomers of (BA)‐**28** could possibly be explained by the ability to fold into the most globular structure, as outlined in **TS‐10**. These preliminary results indicated that cascade cyclization of diepoxides within capsule **15** is possible and promises access to interesting chemo‐ and stereoselectivity. More detailed investigations of this specific topic are ongoing and will be reported in due course.

## Conclusion

Four epoxide‐opening ether cyclizations have been explored as standards for the comparative assessment of catalytic systems that integrate complementary principles from supramolecular chemistry. Supramolecular capsules with π‐basic but a Brønsted acidic inner surface are newly introduced as contrast against general Brønsted and Lewis acids and hydrogen‐bonding, pnictogen‐bonding and anion–π catalysts. They are shown to be as good as pnictogen‐bonding catalysts in violating the Baldwin rules and provide access to interesting selectivities that deserve continuing attention. This is true with regard to the impact of substrate stereochemistry on chemoselectivity, expansion of the substrate collection toward different diepoxides, triepoxides, and longer cascades,[^2^, ^4^] and comparison with other catalyst types, including the halogen‐,[^5^, ^29^] chalcogen‐[^5^, ^14^, ^15^, ^16^, ^30^] and tetrel‐bonding homologs of pnictogen‐bonding[^15^, ^31^] catalysts,[^4^, ^14^] as well as more complex catalytic systems.[^3^, ^31^]

Autocatalysis, however, remains unique for anion–π interactions. The structural space allowed for product‐like co‐catalysts turns out to be intriguingly small, including diastereoselectivity, but not fully sufficient to apply for self‐replication. Moreover, methods to probe for asymmetric anion‐π autocatalysis were introduced. This tantalizing topic,^[21]^ however, is shown not to be a low‐hanging fruit.

## Experimental Section

Please see the Supporting Information.

## Conflict of interest

The authors declare no conflict of interest.

## Supporting information

As a service to our authors and readers, this journal provides supporting information supplied by the authors. Such materials are peer reviewed and may be re‐organized for online delivery, but are not copy‐edited or typeset. Technical support issues arising from supporting information (other than missing files) should be addressed to the authors.

Supporting Information
