# Supplementary material for: Bioinspired Ether Cyclizations within a π‐Basic Capsule Compared to Autocatalysis on π‐Acidic Surfaces and Pnictogen‐Bonding Catalysts
Source: Chemistry. 2021 Jul 8;27(47):12215–23. doi: 10.1002/chem.202101548 (PMC8456975; doi:10.1002/chem.202101548)
Supplement: Supplementary file 1 — Supporting Information [file CHEM-27-12215-s001.pdf]

Chemistry–A European Journal

Supporting Information

Bioinspired Ether Cyclizations within a π-Basic Capsule Compared to Autocatalysis on π-Acidic Surfaces and Pnictogen-Bonding Catalysts

Xiaoyu Hao[+], Tian-Ren Li[+], Hao Chen, Andrea Gini, Xiang Zhang, Stéphane Rosset, Clément Mazet, Konrad Tiefenbacher,* and Stefan Matile*

Table of Contents

1.	Materials and Methods	S3
2.	Synthesis	S4
2.1.	Synthesis of Chiral Tetrahydrofuran Derivative **5**	S4
2.2.	Synthesis of Chiral Tetrahydrofuran Derivative **6**	S6
3.	Kinetics Analysis	S8
4.	Catalysis	S31
4.1.	Co-Catalyst Screening	S31
4.2.	Catalyst Comparison on the Mono-Epoxide Level	S31
4.3.	Catalyst Comparison on the Di-Epoxide Level	S34
4.4.	Asymmetric Autocatalysis Assay	S37
5.	NMR Spectra	S40
6.	Supporting References	S44

1. Materials and Methods

As in ref [S1], Supporting Information. Reagents for synthesis were purchased from Fluka, Sigma-Aldrich, Apollo Scientific and Acros. All solvents used in this study were passed through a 3.0 cm ALOX basic column to remove acidic impurities (such as HCl). Column chromatography was carried out on silica gel (SiliaFlash® P60, SILICYCLE, 230–400 mesh). Analytical (TLC) and preparative thin layer chromatography (PTLC) were performed on silica gel 60 F254 (Merck) and silica gel (SiliCycle, 1000 μm), respectively. Chiral Gas chromatography (GC) was performed on Agilent 6850 Series gas chromatographs equipped with a split-mode capillary injection system and flame ionization detectors using chiral stationary column Hydrodex Gamma DiMOM column (50m x 0.25 mm ID). Separation parameters: 60 °C, 1 °C/min, until 115 °C, then 10 °C/min until 170 °C (Speed: 60 cm/s H_2, injector temperature: 170 °C). ^{1}H and ^{13}C NMR were recorded (as indicated) either on a Bruker 300 MHz or 400 MHz spectrometer and are reported as chemical shifts (δ) in ppm relative to TMS ($\delta = 0$). Spin multiplicities are reported as a singlet (s), doublet (d), triplet (t) and quartet (q), with coupling constants (J) given in Hz, or multiplet (m). Broad peaks are marked as br.

Abbreviations. DMAP: 4-Dimethylaminopyridine; DMM: Dimethoxymethane; EDC: 1-Ethyl-3-(3-dimethylaminopropyl)carbodiimide; rt: Room temperature; TBAF: Tetra-*n*-butylammonium fluoride; TES: Tetraethylsilane; TESOTf: Trimethylsilyl trifluoromethanesulfonate.

2. Synthesis

Compound 1 was prepared following previously reported procedures.[S2]

Compound 2 was prepared following previously reported procedures.[S3]

Compound 3 was prepared following previously reported procedures.[S4]

Compounds 4-6, 9, 13-14, 26 were prepared following previously reported procedures.[S5]

Compound 11 was prepared following previously reported procedures.[S6]

Compound 12 was prepared following previously reported procedures.[S7]

Compound 16 was prepared following previously reported procedures.[S8]

Compound (*S*)-**24** was prepared following previously reported procedures.[S9]

2.1. Synthesis of Chiral Tetrahydrofuran Derivative 5

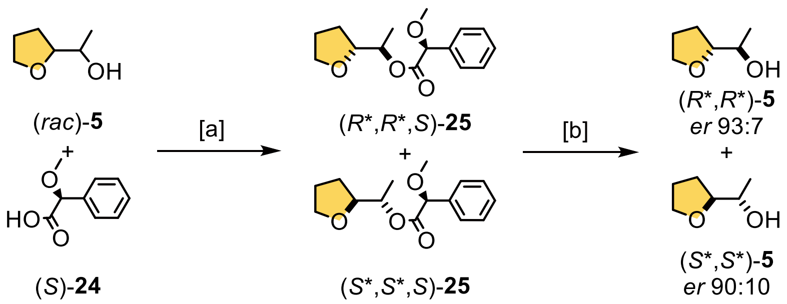

Scheme S1. [a] EDC, DMAP, CH_2Cl_2, 30% + 37%. [b] K_2CO_3, MeOH, 30% and 20%.

Compound (*R,*R**,*S*)-25 and Compound (*S**,*S**,*S*)-25:** To a solution of (*rac*)-**5** (10 mg, 0.10 mmol) in CH_2Cl_2 (1.0 mL), DMAP (15 mg, 0.12 mmol) and (*S*)-**24** (16 mg, 0.10 mmol) were added. Then, EDC•HCl (24 mg, 0.12 mmol) was added portion by portion at rt. The mixture was stirred at rt for 15 h. Then, the reaction mixture was purified by silica gel column chromatography

directly (pentane/EtOAc 10:1 to 5:1) to give (*R**,*R**,*S*)-**25** (8.0 mg, 30% yield) and (*S**,*S**,*S*)-**25** (10 mg, 37% yield, (*S**,*S**,*S*)-**25** : (*R**,*R**,*S*)-**25** = 2:1) both as colorless oil. Note, the configurations (*R**,*R**) and (*S**,*S**) are arbitrarily assigned.

Compound (*R,*R**,*S*)-25:** R_f (pentane/EtOAc 2:1) = 0.5; ^{1}H NMR (300 MHz, $CDCl_3$): 7.49 – 7.42 (m, 2H), 7.41 – 7.32 (m, 3H), 5.00 – 4.90 (m, 1H), 4.78 (s, 1H), 3.88 – 3.76 (m, 1H), 3.74 – 3.59 (m, 2H), 3.41 (s, 3H), 1.79 – 1.46 (m, 3H), 1.29 (d, J = 6.4 Hz, 3H), 1.24 – 1.05 (m, 1H); ^{13}C NMR (75 MHz, $CDCl_3$): 170.5 (C), 136.5 (C), 128.7 (CH), 128.6 (CH), 127.4 (CH), 82.7 (CH), 80.2 (CH), 72.8 (CH), 68.4 (CH_2), 57.3 (CH_3), 27.3 (CH_2), 25.8 (CH_2), 16.7 (CH_3).

Compound (*S,*S**,*S*)-25:** R_f (pentane/EtOAc 2:1) = 0.4; ^{1}H NMR (300 MHz, $CDCl_3$): 7.51 – 7.42 (m, 2H), 7.41 – 7.31 (m, 3H), 5.04 – 4.87 (m, 1H), 4.79 (s, 1H), 3.92 – 3.64 (m, 3H), 3.43 (s, 3H), 1.90 – 1.46 (m, 2H), 1.71 – 1.46 (m, 2H), 1.06 (d, J = 6.4 Hz, 3H); ^{13}C NMR (75 MHz, $CDCl_3$): 170.4 (C), 136.4 (C), 128.6 (CH), 127.4 (CH), 127.2 (CH), 82.5 (CH), 80.4 (CH), 73.3 (CH), 68.4 (CH_2), 57.3 (CH_3), 27.9 (CH_2), 25.9 (CH_2), 16.1 (CH_3).

Compound (*R,*R**)-5 and Compound (*S**,*S**)-5:** To a solution of (*R**,*R**,*S*)-**25** or (*S**,*S**,*S*)-**25** (140 mg, 0.530 mmol) in MeOH (5.0 mL), K_2CO_3 (290 mg, 2.10 mmol) was added at room temperature. The reaction mixture was stirred for 15 h. The reaction mixture was filtered and rinsed with CH_2Cl_2 (5.0 mL). The filtrate was concentrated and purified by silica gel column chromatography (pentane/EtOAc 2:1 to 1:1) to give (*R**,*R**)-**5** (12 mg, 20% yield) or (*S**,*S**)-**5** (20 mg, 30% yield) as colorless oil. R_f (pentane/EtOAc 2:1) = 0.2. Note, the configurations (*R**,*R**) and (*S**,*S**) are arbitrarily assigned.

Compound (*R,*R**)-5 (86% ee):** $[\alpha]_D^{20}$ +9.2 (*c* 0.24, $CDCl_3$)

Compound (*S,*S**)-5 (80% ee):** $[\alpha]_D^{20}$ -2.7 (*c* 0.94, $CDCl_3$)

2.2. Synthesis of Chiral Tetrahydrofuran Derivative 6

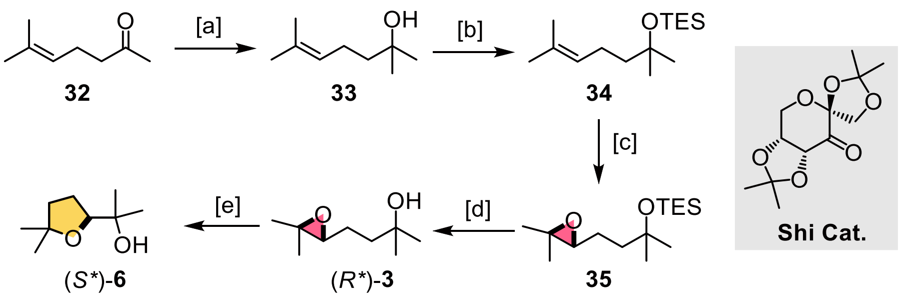

Scheme S2. [a] MeMgBr, THF, 0 ºC, 80%.[S10] [b] TESOTf, Et_3N, CH_2Cl_2, rt, 95%. [c] **Shi Cat.**, Oxone, K_2CO_3, $Na_2B_4O_7$/*n*-Bu_4NHSO_4 buffer, DMM/MeCN, 0 ºC, 94%. [d] TBAF, THF, rt, 0 ºC, 65%. [e] AcOH, CH_2Cl_2, rt, 66%.

Compound 33 was prepared following previously reported procedures.[S10]

Compound 34: To a solution of **33** (0.80 g, 5.6 mmol) in CH_2Cl_2 (10 mL), TESOTf (1.4 mL, 6.7 mmol) and NEt_3 (0.90 mL, 6.7 mmol) were added. The reaction mixture was stirred for 23 h at rt. Then, it was quenched by water (20 mL), extracted by CH_2Cl_2 (3 x 20 mL). The organic phase was combined, concentrated, dried over Na_2SO_4 and purified by silica gel column chromatography (pentane/EtOAc 100:1) to give **34** (1.43 g, 95% yield) as colorless oil. R_f (pentane/EtOAc 50:1) = 0.5; ^{1}H NMR (300 MHz, $CDCl_3$): 5.12 (t, J = 7.2 Hz, 1H), 2.08 – 2.01 (m, 2H), 1.69 (s, 3H), 1.62 (s, 3H), 1.52 – 1.39 (m, 2H), 1.21 (s, 6H), 0.96 (t, J = 7.8 Hz, 9H), 0.58 (q, J = 7.8 Hz, 6H); ^{13}C NMR (75 MHz, $CDCl_3$): 131.0 (C), 125.0 (CH), 73.2 (C), 45.0 (CH_2), 29.8 (CH_3), 25.7 (CH_3), 23.1 (CH_3), 17.6 (CH_2), 7.1 (CH_2), 6.8 (CH_3).

Compound 35: Compound **34** (184 mg, 1 mmol) was dissolved in a 1:2 v/v mixture of acetonitrile:dimethoxymethane (45 mL). A 0.05 M solution of $Na_2B_4O_7\cdot10H_2O$ (in 4×10^{-4} M aqueous solution of Na_2EDTA, 30 mL), *n*-Bu_4NHSO_4 (45 mg, 0.12 mmol) and **Shi Cat.** (0.38 g, 1.5 mmol)

were sequentially added under vigorous stirred at 0 °C. To this mixture a solution of Oxone (2.7 g, 4.5 mmol, in 4×10^{-4} M aqueous solution of Na_2EDTA, 21 mL), and K_2CO_3 (2.6 g, 18 mmol, dissolved in water (21 mL)), were simultaneously added over 2 h via syringe pump. At this point, the mixture was quenched with water (20 mL), extracted with CH_2Cl_2 (3 x 20 mL), washed with brine, dried over Na_2SO_4, purified by flash column chromatography (pentane/EtOAc 30:1) to give **35** (0.8 g, 94% yield) as colorless oil. R_f (pentane/EtOAc 10:1) = 0.5; ^{1}H NMR (300 MHz, $CDCl_3$): 2.72 (t, J = 6.0 Hz, 1H), 1.69 – 1.59 (m, 3H), 1.57 – 1.42 (m, 1H), 1.32 (s, 3H), 1.28 (s, 3H), 1.23 (s, 3H), 1.22 (s, 3H), 0.95 (t, J = 7.9 Hz, 9H), 0.58 (q, J = 7.9 Hz, 6H); ^{13}C NMR (75 MHz, $CDCl_3$): 72.9 (C), 64.7 (CH), 58.3 (C), 41.4 (CH_2), 30.0 (CH_3), 29.6 (CH_3), 25.0 (CH_3), 24.0 (CH_3), 18.6 (CH_2), 7.1 (CH_2), 6.8 (CH_3).

Compound (*R)-3:** To a solution of **35** (0.80 g, 2.9 mmol) in THF (3 mL), TBAF (3.0 mL, 1.0 M in THF) was added. The reaction mixture was stirred for 2 h at rt and purified by flash column chromatography directly (pentane/EtOAc 2:1 to 1:1) to give (*R**)-**3** (0.3 g, 65% yield) as colorless oil; R_f (EtOAc) = 0.5.

Compound (*S)-6:** To a solution of (*R**)-**3** (0.30 g, 1.9 mmol) in CH_2Cl_2 (5 mL), AcOH (1.1 mL, 19 mmol) was added. The reaction mixture was stirred at rt for 24 h. Then, it was diluted with CH_2Cl_2 (20 mL), the organic phase was washed with brine, dried over Na_2SO_4, and purified by flash column chromatography (pentane/EtOAc 4:1) to give (*S**)-**6** (0.2 g, 66% yield) as colorless oil; R_f (pentane/EtOAc 1:1) = 0.6.

Compound (*S)-6 (79% ee):**[S11] $[\alpha]_D^{20}$ -5.1 (*c* 0.49, $CDCl_3$)

3. Kinetics Analysis

*Procedures for anion-π catalyst **10**.*

General procedure A: Solutions of substrate **1**, **2** or **3** (1 M) and internal standard dibromomethane (140 mM) and different amount of the corresponding product **4**, **5**, **6** or **9** were mixed in **10**, and stirred at 20 °C. ^{1}H NMR spectra of the mixture diluted in CD_2Cl_2 were recorded at varying time intervals.

Kinetic studies: Concentrations of products were estimated from the consumption of the substrate and were plotted against time. Here pseudo-first-order conditions are assumed for the analysis of the autocatalysis,[S12] and the reaction rate (r) can be expressed as

$$r = k_1[\mathrm{R}] + k_2[\mathrm{R}][\mathrm{P}] \qquad [S1]$$

where k_1 and k_2 are the rate constants corresponding to the non(auto)catalytic and the (auto)catalytic mechanisms, respectively. Assuming first order in both reactant (R) and autocatalytic product (P), and

$$[\mathrm{P}] = [\mathrm{R}]_0 - [\mathrm{R}] \qquad [S2]$$

then,

$$[\mathrm{P}] = [\mathrm{R}]_0 \times \left(1 - \frac{b + k_1}{b + k_1 \exp{(k_1 + b)t}}\right) \qquad [S3]^{[S13]}$$

where,

$$b = [\mathrm{R}]_0 k_2 \qquad [S4]$$

The rate constants k_1 and k_2 were obtained by fitting the data to the equations S3 and S4. The substrate half-lifetimes (t_{50}) were obtained using Equation [S5].

$$t_{50} = \ln(b/k_1+2)/(b+k_1) \quad [S5]$$

*Procedures for capsule **15**.*

General procedure B: To a 5 mm NMR tube, substrate **1**, **2** or **3** (33.3 mM) and different amount of the corresponding product **4**, **5**, **6** or **9** were dissolved into 500 µL $CDCl_3$ (pre-treated as described in the Materials and Methods section). 10.0 µL TES stock solution (prepared by dissolving 40.0 µL TES into 1.00 mL $CDCl_3$. This stock solution was stored in -20 ºC freezer and warmed to room temperature before use) was injected as internal standard. After that, capsule **15** (11 mg, 10 mol%) was added and the reaction was monitored by ^{1}H NMR.

Kinetic studies: The pseudo-first-order rate constant (k) was estimated by fitting the data to the Equation [S6]:

$$[P] = [R]_0 - ([R]_0 - [P]_0)\cdot\exp(-kt) \quad [S6]$$

[P] starts at $[P]_0 = 0$, then goes up to $[R]_0$ with one phase. The rate constants k was obtained by fitting the data to Equation [S6]. The substrate half-lifetimes (t_{50}) were obtained using Equation [S7].

$$t_{50} = \ln[2\cdot([R]_0 - [P]_0)/[R]_0]/k \quad [S7]$$

Table S1. Kinetics analysis for substrate **1** with and without co-catalyst (B)-**4**.[a]

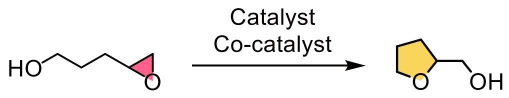

1 (B)-**4**

Cat[b]	Co-cat / mol%[c]	k_{non} / h^{-1}[d]	t_{50} / h[e]	k_{auto} / M^{-1} h^{-1}[f]	k / h^{-1}[g]
**10**	0	$(2.5 \pm 1.6) \times 10^{-3}$	>> 10	0.47 ± 0.14	-
**10**	50	$(3.0 \pm 1.1) \times 10^{-2}$	5.23 ± 0.16	0.54 ± 0.10	-
**10**	100	$(3.0 \pm 0.3) \times 10^{-2}$	3.96 ± 0.11	0.82 ± 0.04	-
**10**	200	$(7.4 \pm 2.9) \times 10^{-2}$	2.80 ± 0.14	0.86 ± 0.21	-
**15**	0	-	10.8 ± 0.8	-	$(6.4 \pm 0.4) \times 10^{-2}$

[a] See Figure 3 in manuscript, conditions: 1.0 M (with **10**) or 33.3 mM (with **15**) substrate, rt. [b] Catalyst, **10** = solvent, **15**: 10 mol% in $CDCl_3$. [c] Co-catalyst: (B)-**4**. [d] Non-autocatalytic rate constant. [e] Reaction half-life time. [f] Autocatalytic rate constant. [g] Catalytic rate constant.

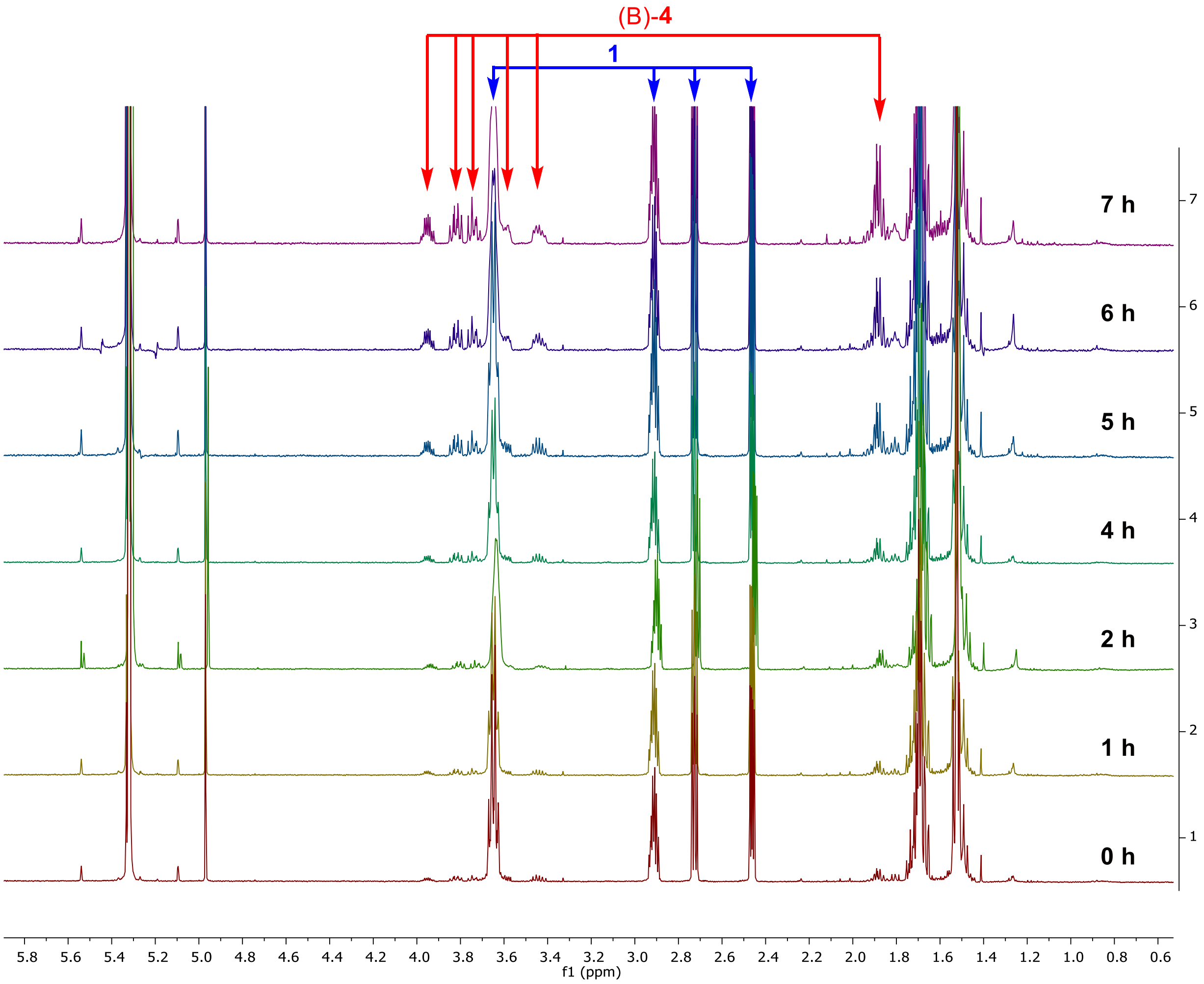

Figure S1. ^{1}H NMR spectra of reaction kinetics at room temperature for the conversion of **1** into (B)-**4** in the presence of anion-π catalyst **10** in CD_2Cl_2.

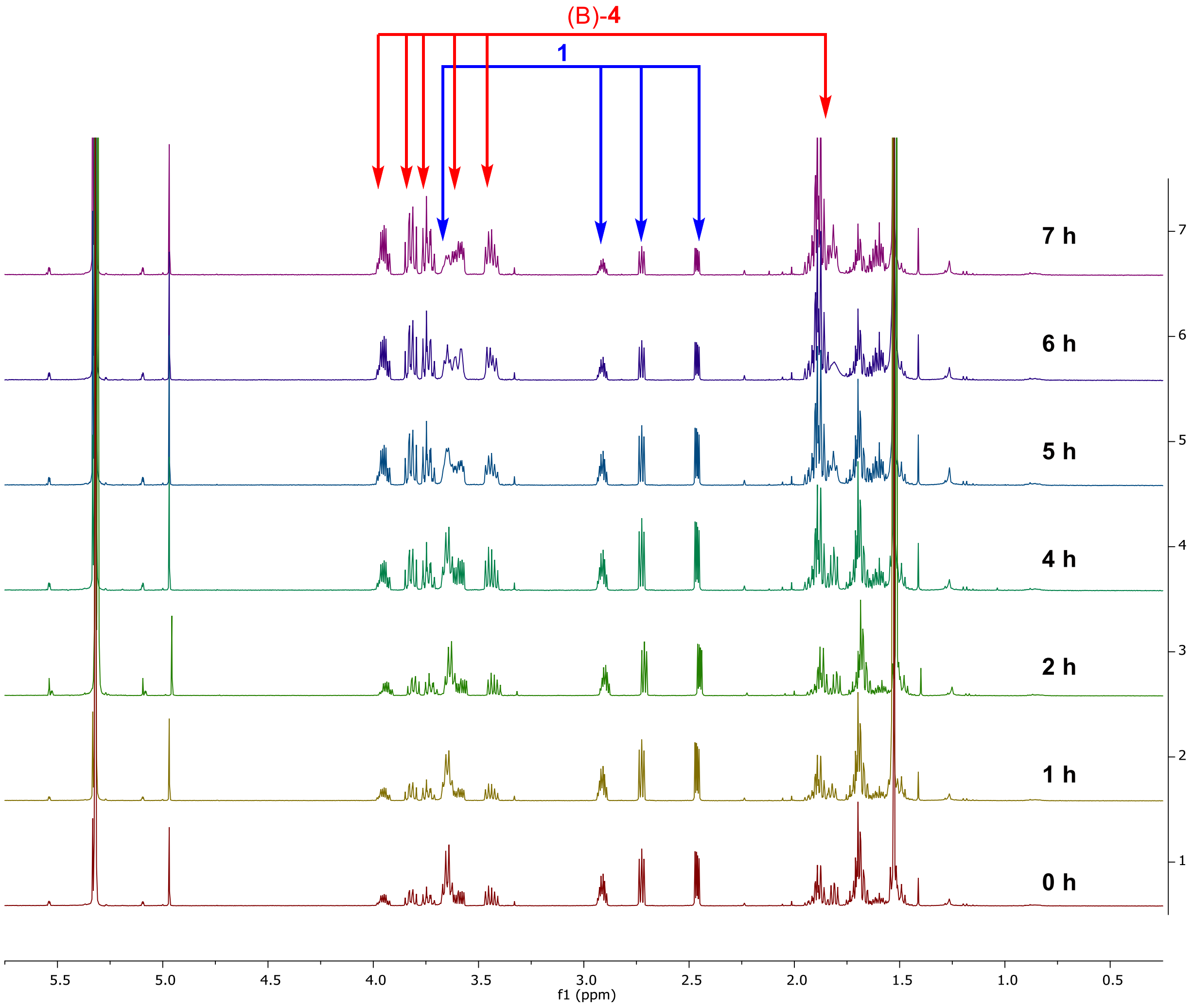

Figure S2. ^{1}H NMR spectra of reaction kinetics at room temperature for the conversion of **1** into (B)-**4** in the presence of anion-π catalyst **10** and 0.5 equivalents of **4** added at the beginning of the reaction in CD_2Cl_2.

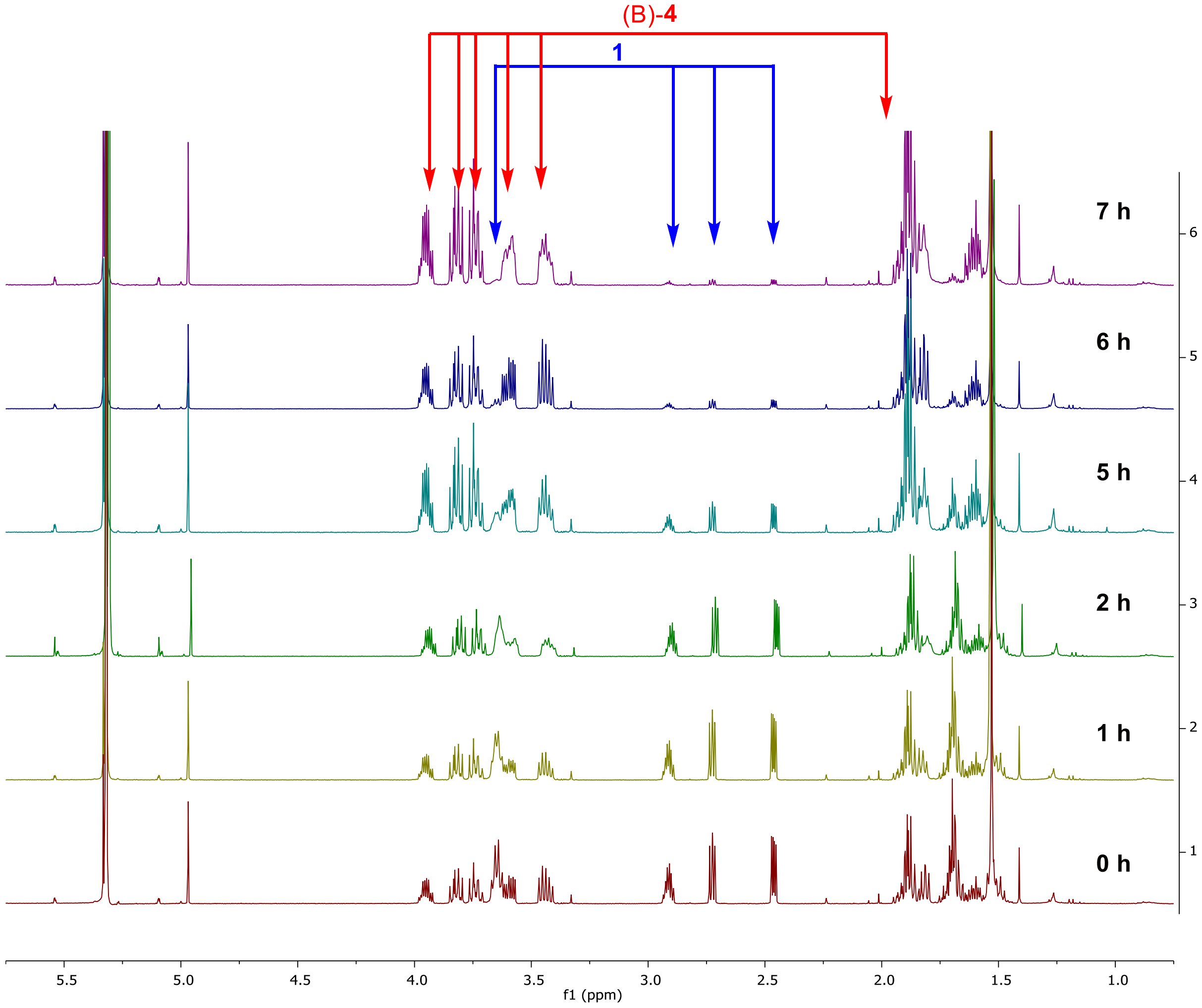

Figure S3. ^{1}H NMR spectra of reaction kinetics at room temperature for the conversion of **1** into (B)-**4** in the presence of anion-π catalyst **10** and 1.0 equivalents of **4** added at the beginning of the reaction in CD_2Cl_2.

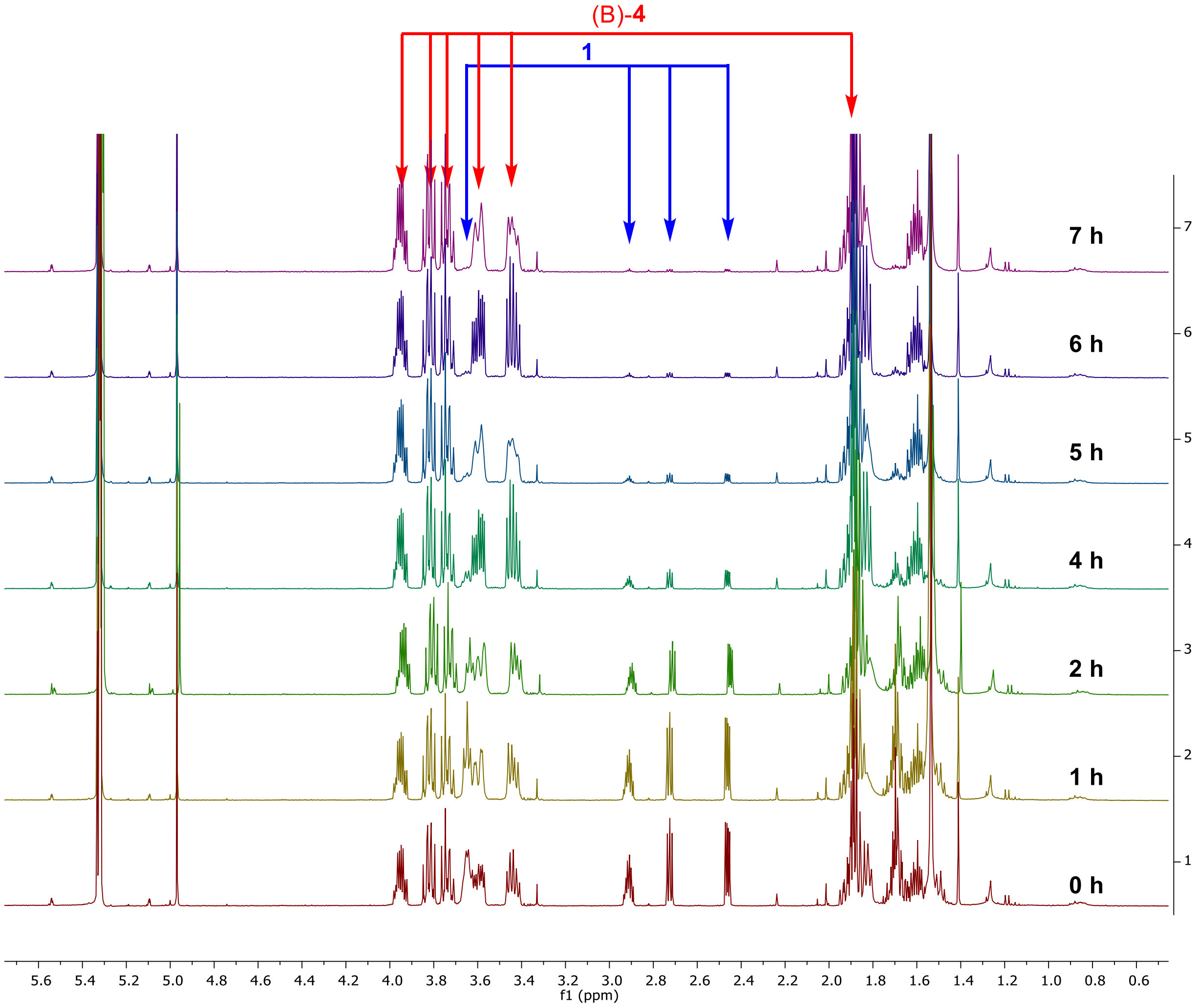

Figure S4. ^{1}H NMR spectra of reaction kinetics at room temperature for the conversion of **1** into (B)-**4** in the presence of anion-π catalyst **10** and 2.0 equivalents of **4** added at the beginning of the reaction in CD_2Cl_2.

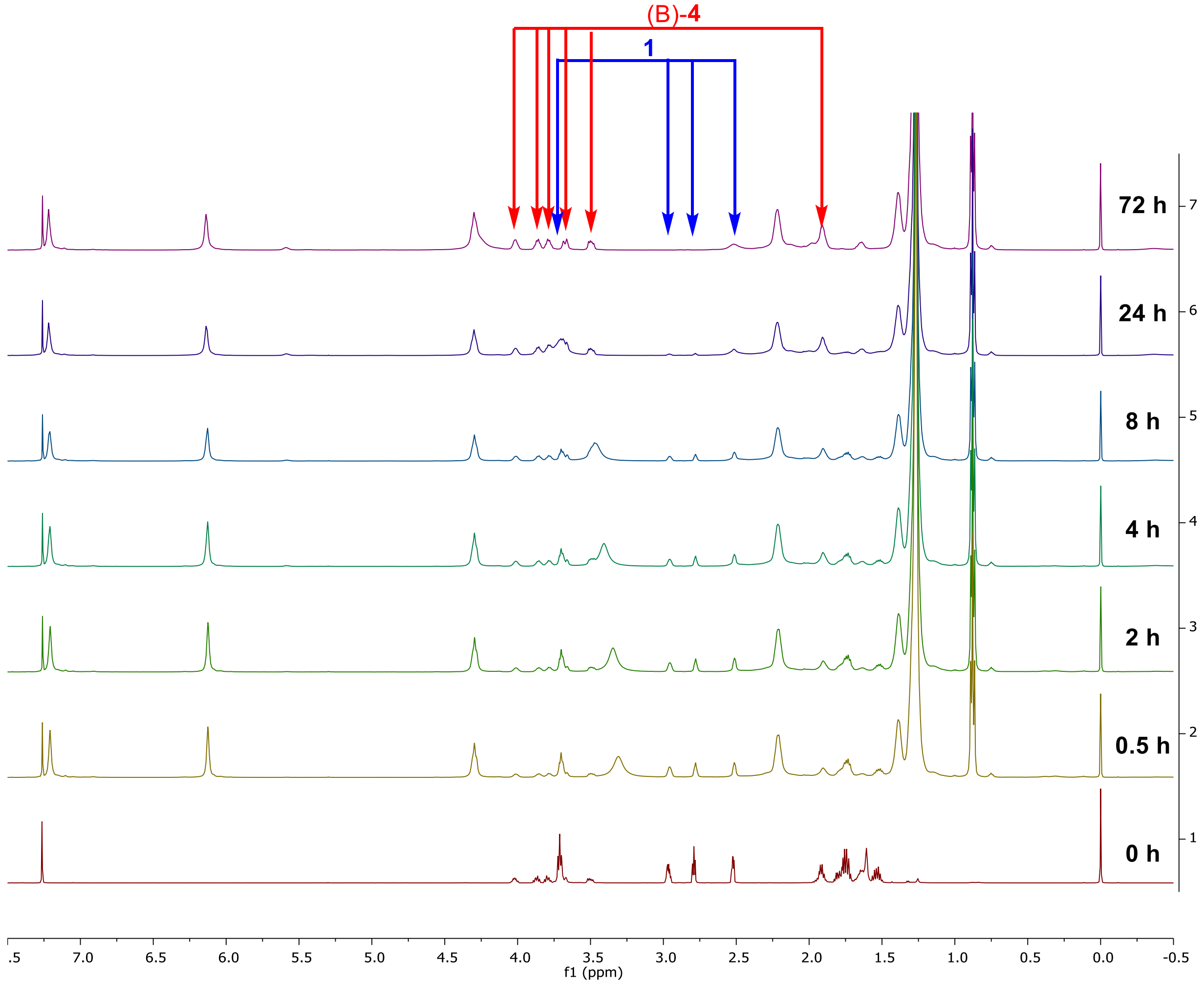

Figure S5. ^{1}H NMR spectra of reaction kinetics at room temperature for the conversion of **1** into (B)-**4** in the presence of capsule **15** in $CDCl_3$.

Table S2. Kinetics analysis for substrate **2** with and without co-catalyst (B)-**5**.[a]

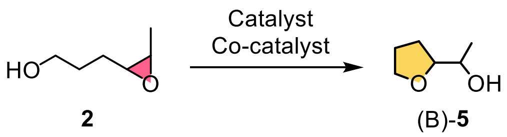

Cat[b]	Co-cat / mol%[c]	k_{non} / h^{-1}[d]	t_{50} / h[e]	k_{auto} / M^{-1} h^{-1}[f]	k / h^{-1}[g]
**10**	0	(1.4 ± 0.8) x 10^{-2}	2.56 ± 0.05	1.90 ± 0.25	-
**15**	0	-	0.92 ± 0.12	-	0.75 ± 0.10
**15**	25	-	1.07 ± 0.11	-	0.65 ± 0.07
**15**	50	-	1.45 ± 0.13	-	0.48 ± 0.04
**15**	100	-	1.55 ± 0.11	-	0.45 ± 0.03

[a] See Figure 4a-b in manuscript, conditions: 1.0 M (with **10**) or 33.3 mM (with **15**) substrate, rt. [b] Catalyst, **10** = solvent, **15**: 10 mol% in $CDCl_3$. [c] Co-catalyst: (B)-**5**. [d] Non-autocatalytic rate constant. [e] Reaction half-life time. [f] Autocatalytic rate constant. [g] Catalytic rate constant.

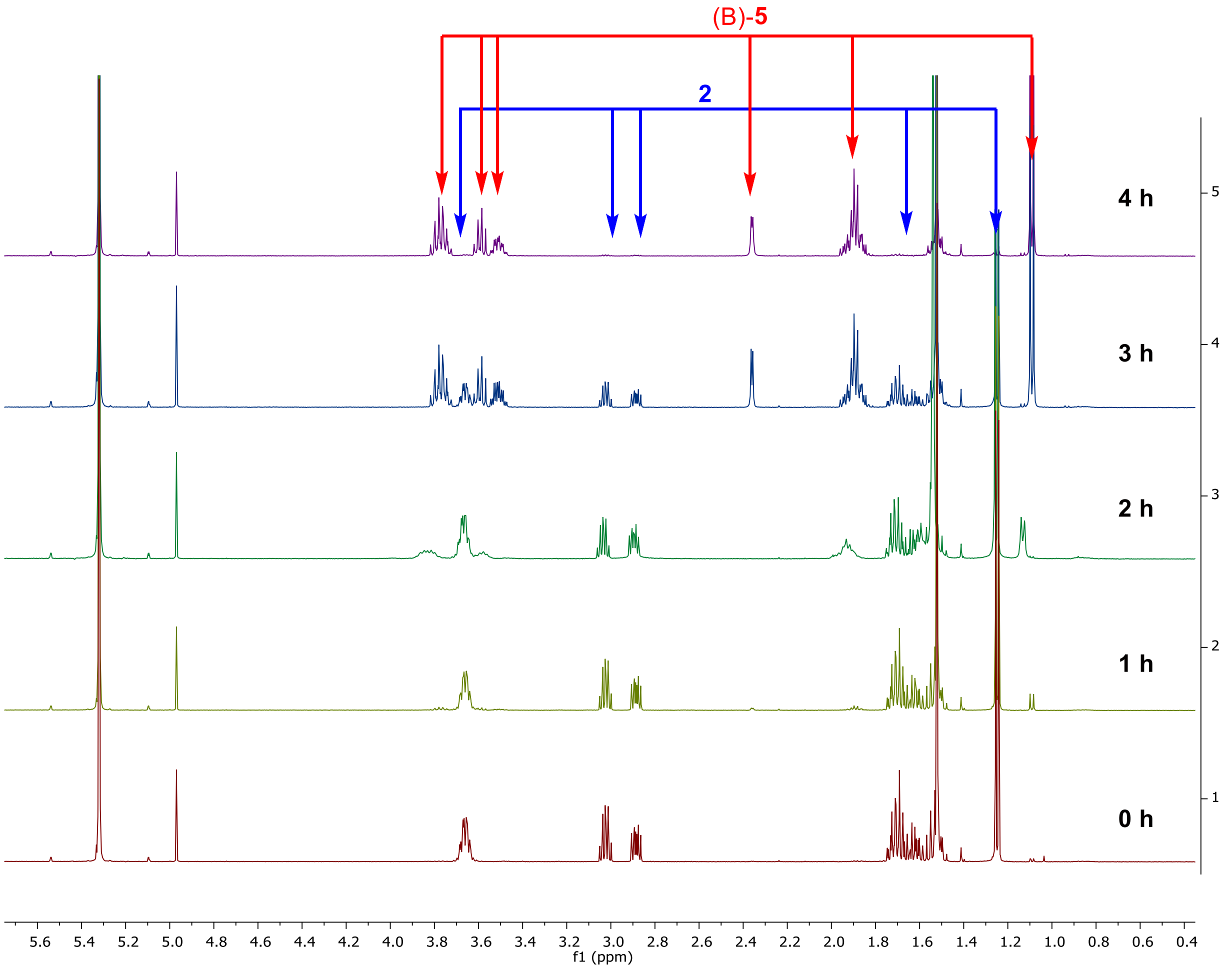

Figure S6. ^{1}H NMR spectra of reaction kinetics at room temperature for the conversion of **2** into (B)-**5** in the presence of anion-π catalyst **10** in CD_2Cl_2.

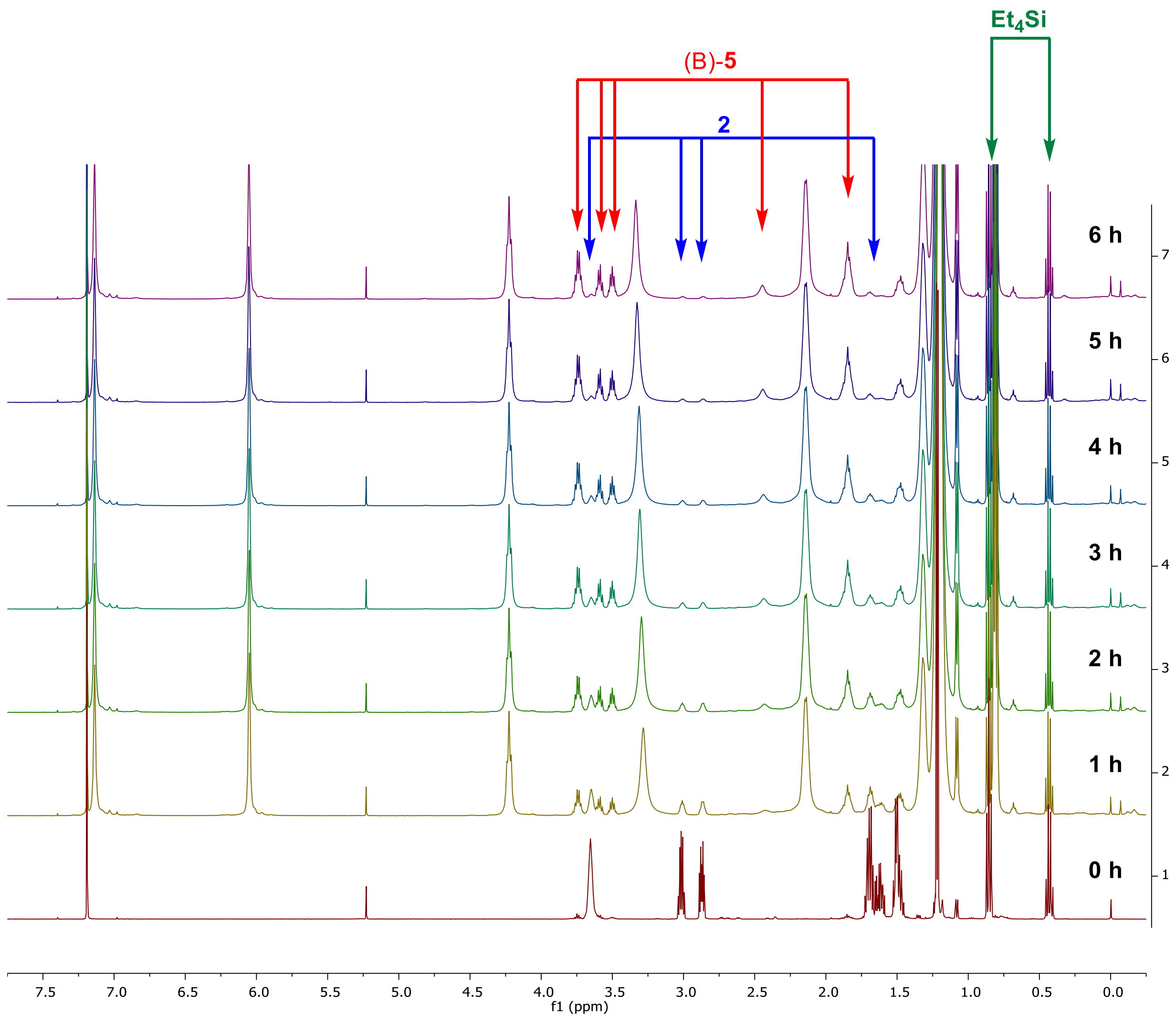

Figure S7. ^{1}H NMR spectra of reaction kinetics at room temperature for the conversion of **2** into (B)-**5** in the presence of capsule **15** in $CDCl_3$.

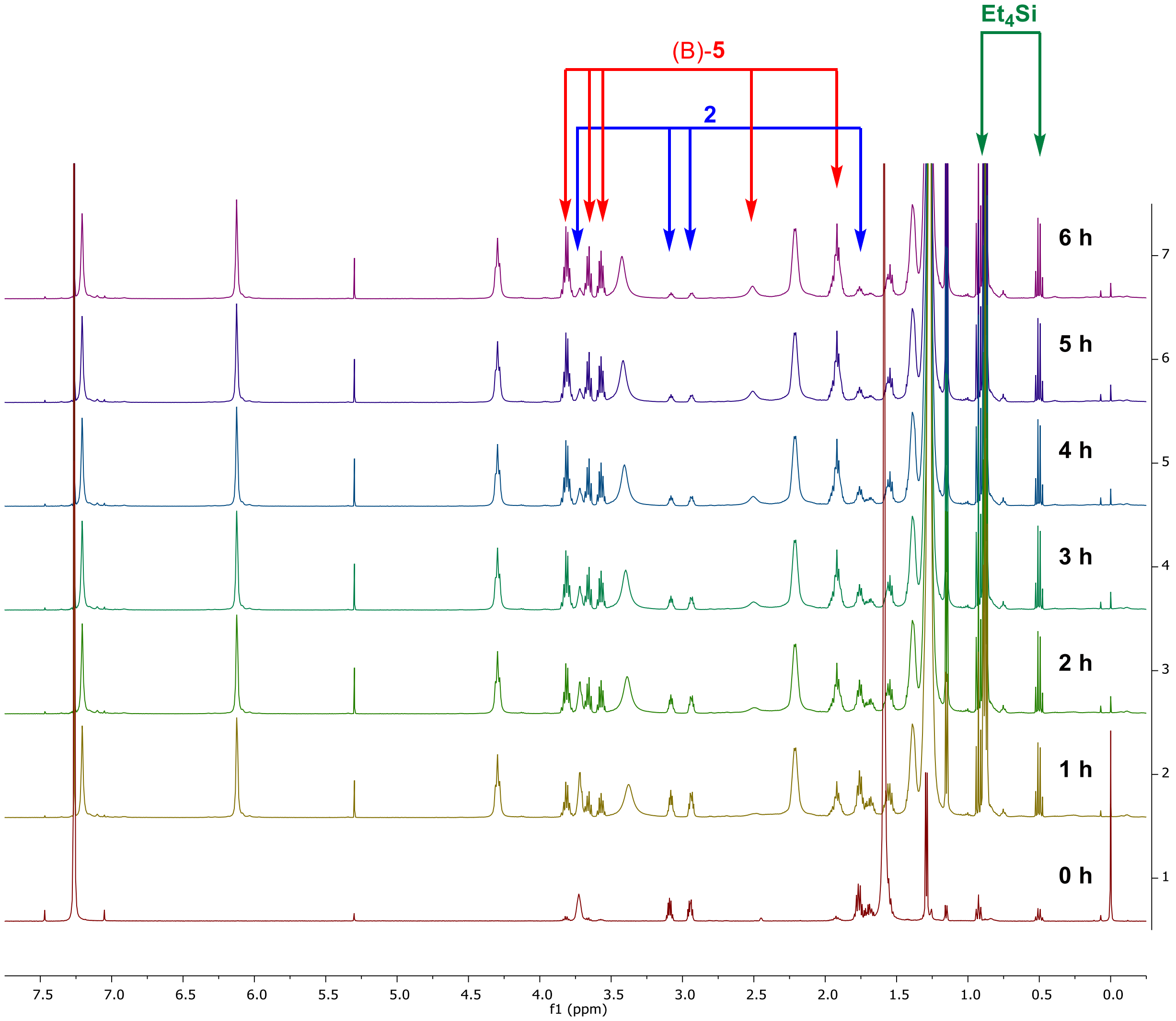

Figure S8. ^{1}H NMR spectra of reaction kinetics at room temperature for the conversion of **2** into (B)-**5** in the presence of capsule **15** and 0.25 equivalents of **5** added at the beginning of the reaction in $CDCl_3$.

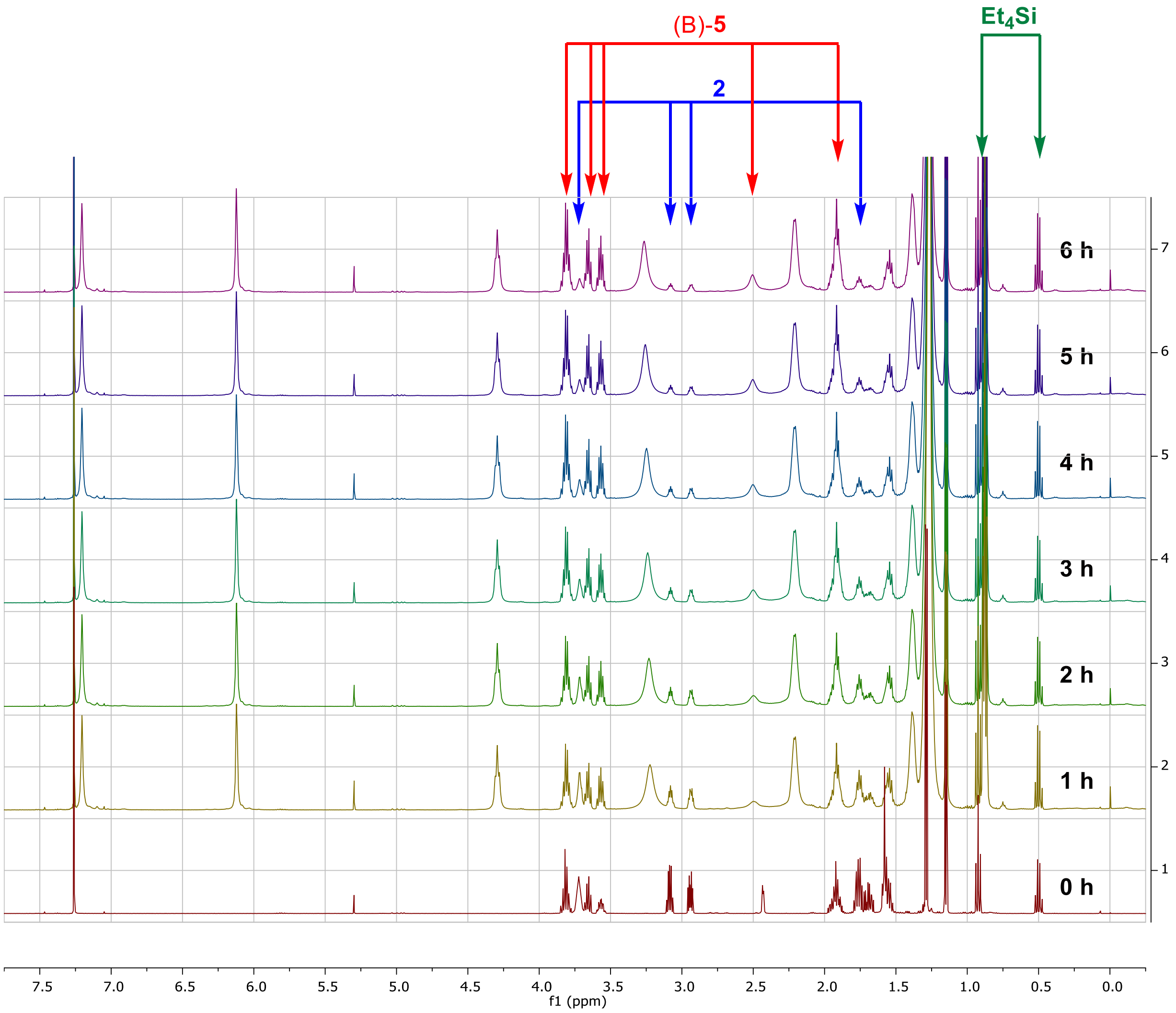

Figure S9. ^{1}H NMR spectra of reaction kinetics at room temperature for the conversion of **2** into (B)-**5** in the presence of capsule **15** and 0.5 equivalents of **5** added at the beginning of the reaction in $CDCl_3$.

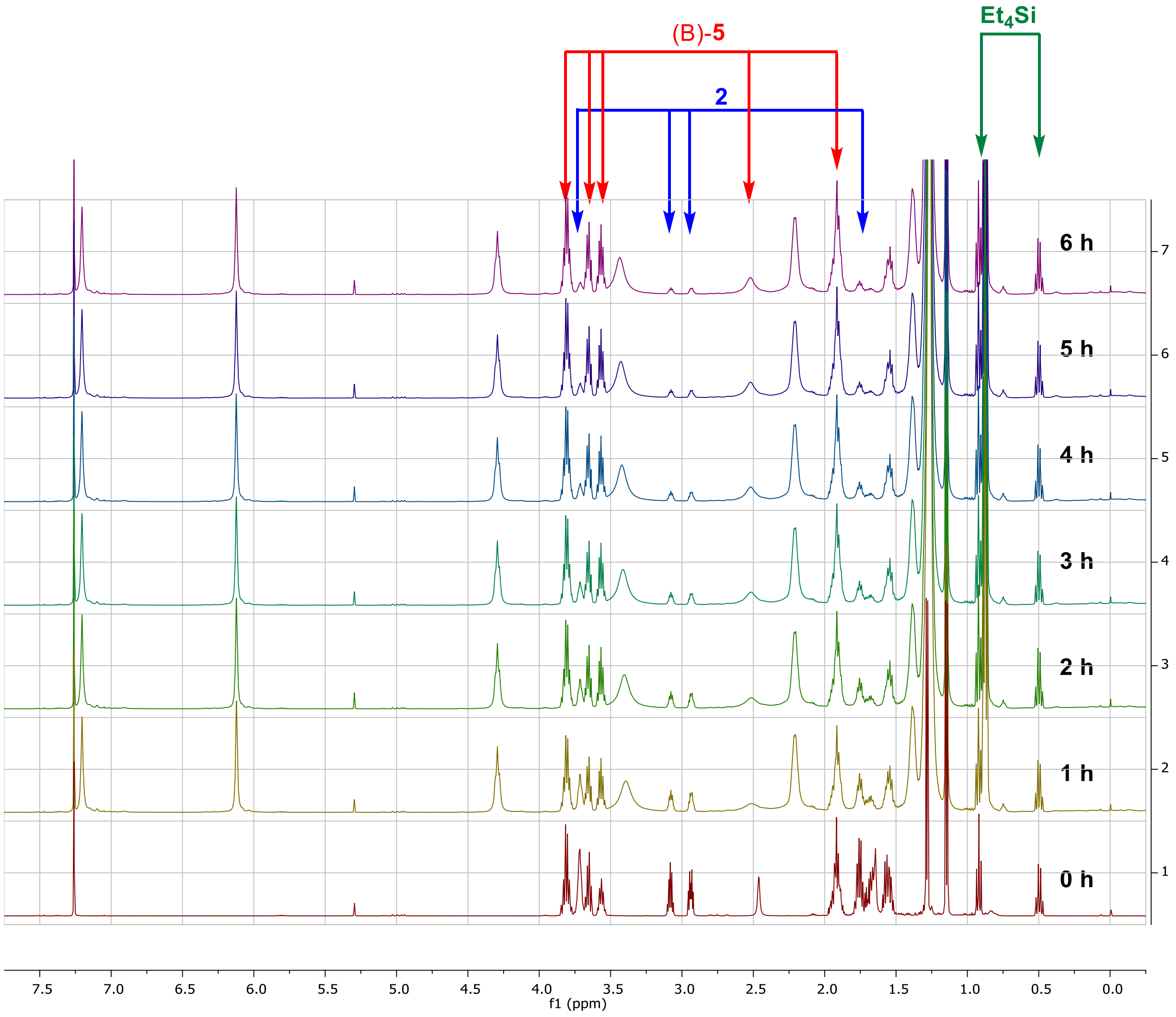

Figure S10. ^{1}H NMR spectra of reaction kinetics at room temperature for the conversion of **2** into (B)-**5** in the presence of capsule **15** and 1.0 equivalents of **5** added at the beginning of the reaction in $CDCl_3$.

Table S3. Kinetics analysis for substrate **3** with and without co-catalyst (B)-**6**.[a]

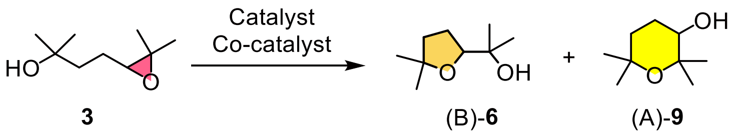

Cat[b]	Co-cat / mol%[c]	k_{non} / h^{-1}[d]	t_{50} / h[e]	k_{auto} / M^{-1} h^{-1}[f]	k / h^{-1}[g]
**10**	0	(1.4 ± 0.5) x 10^{-2}	>> 10	0.15 ± 0.08	-
**10**	10	(6.6 ± 0.8) x 10^{-2}	>> 10	-0.03 ± 0.04	-
**10**	100	(7.1 ± 0.7) x 10^{-2}	4.64 ± 0.20	0.35 ± 0.04	-
**15**	0	-	0.70 ± 0.03	-	0.98 ± 0.05
**15**	25	-	0.55 ± 0.06	-	1.25 ± 0.14
**15**	100	-	0.68 ± 0.07	-	1.01 ± 0.11
**15**	25[h]	-	1.44 ± 0.10	-	0.48 ± 0.03
**15**	100[h]	-	1.77 ± 0.09	-	0.39 ± 0.02

[a] See Figure 6a, b and d in manuscript, conditions: 1.0 M (with **10**) or 33.3 mM (with **15**) substrate, rt. [b] Catalyst, **10** = solvent, **15**: 10 mol% in $CDCl_3$. [c] Co-catalyst: (B)-**6**. [d] Non-autocatalytic rate constant. [e] Reaction half-life time. [f] Autocatalytic rate constant. [g] Catalytic rate constant. [h] (A)-**9** was used instead of (B)-**6**.

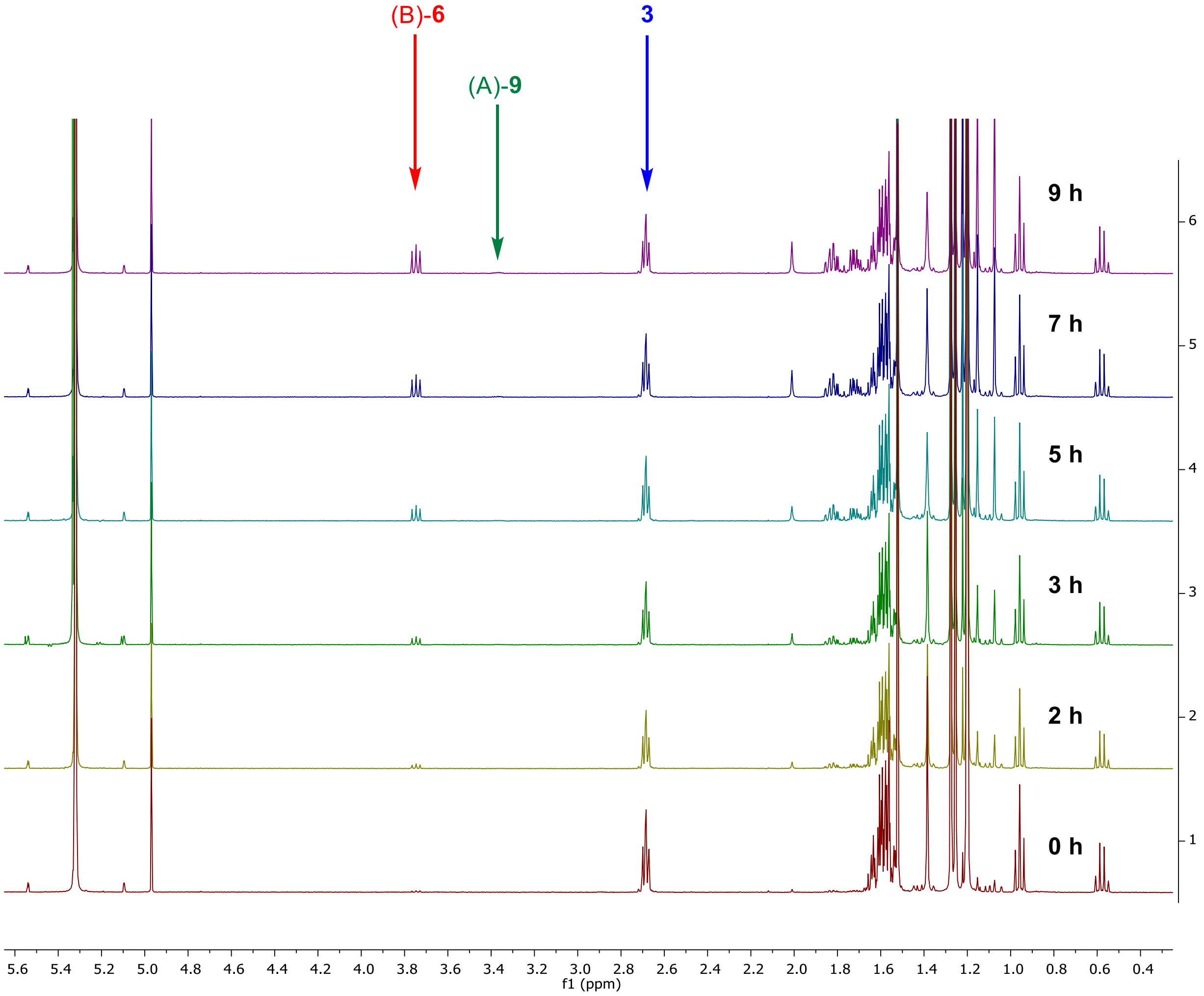

Figure S11. ^{1}H NMR spectra of reaction kinetics at room temperature for the conversion of **3** into (B)-**6** and (A)-**9** in the presence of anion-π catalyst **10** in CD_2Cl_2.

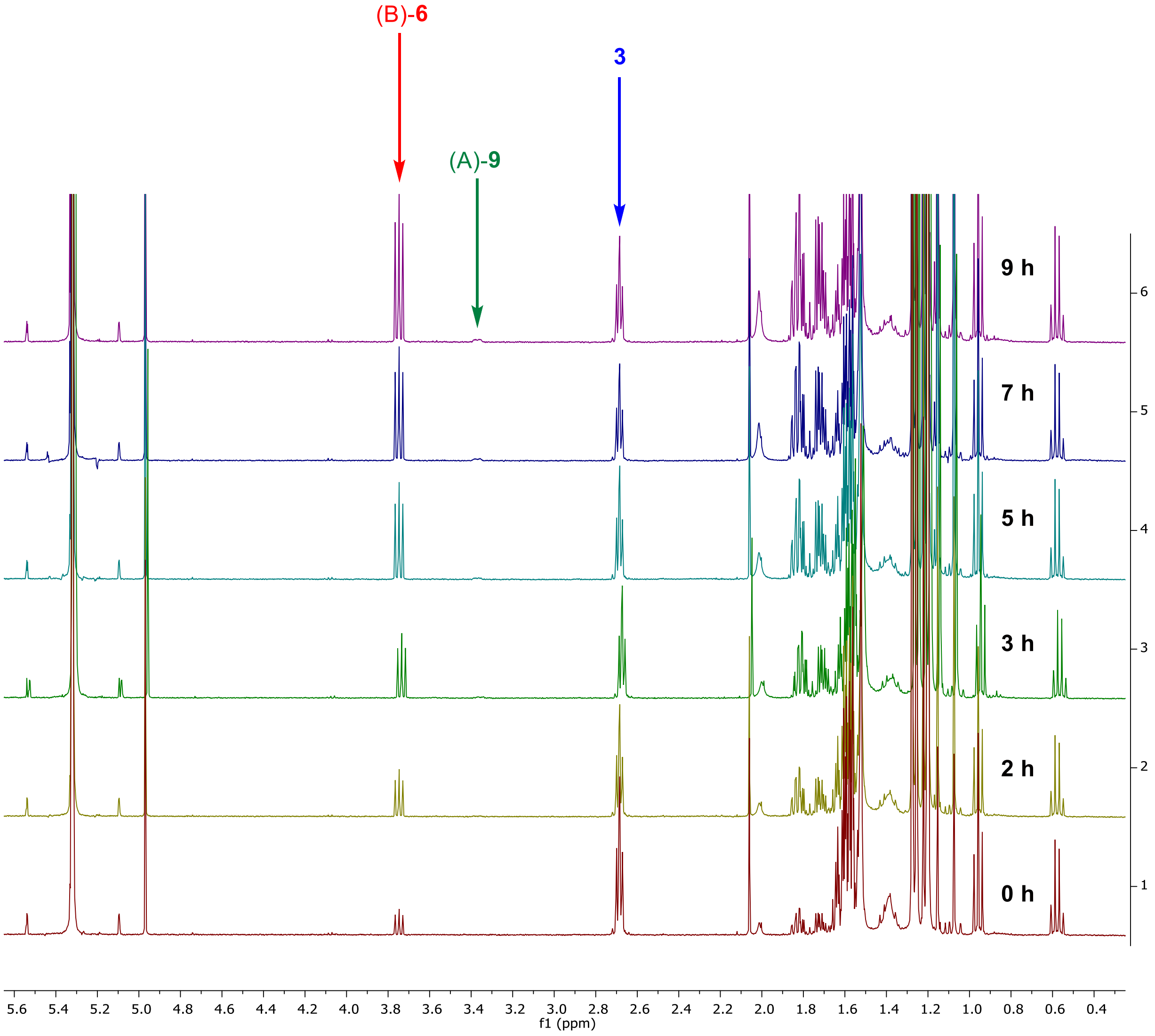

Figure S12. ^{1}H NMR spectra of reaction kinetics at room temperature for the conversion of **3** into (B)-**6** and (A)-**9** in the presence of anion-π catalyst **10** and 0.1 equivalents of **6** added at the beginning of the reaction in CD_2Cl_2.

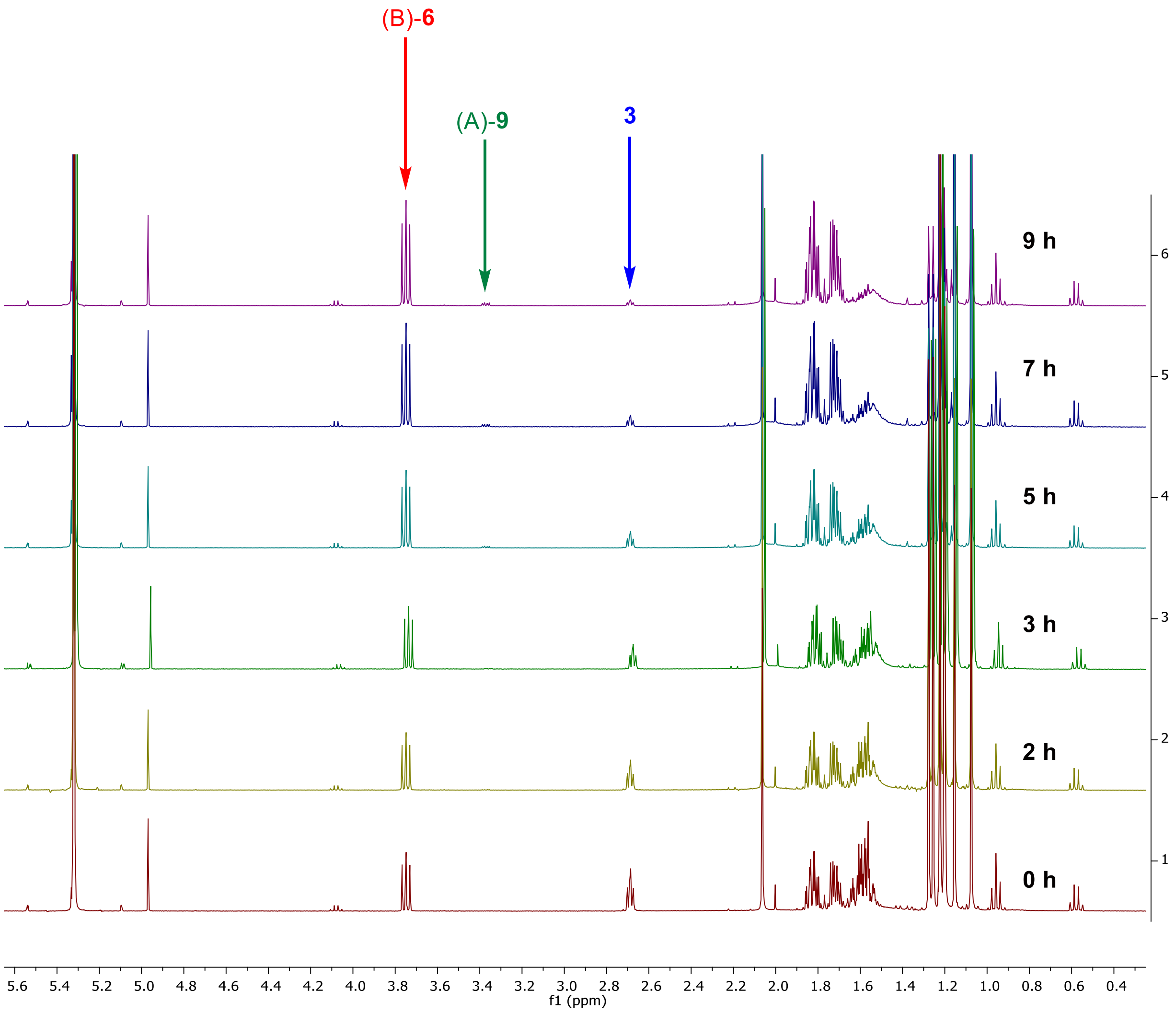

Figure S13. ^{1}H NMR spectra of reaction kinetics at room temperature for the conversion of **3** into (B)-**6** and (A)-**9** in the presence of anion-π catalyst **10** and 1.0 equivalents of **6** added at the beginning of the reaction in CD_2Cl_2.

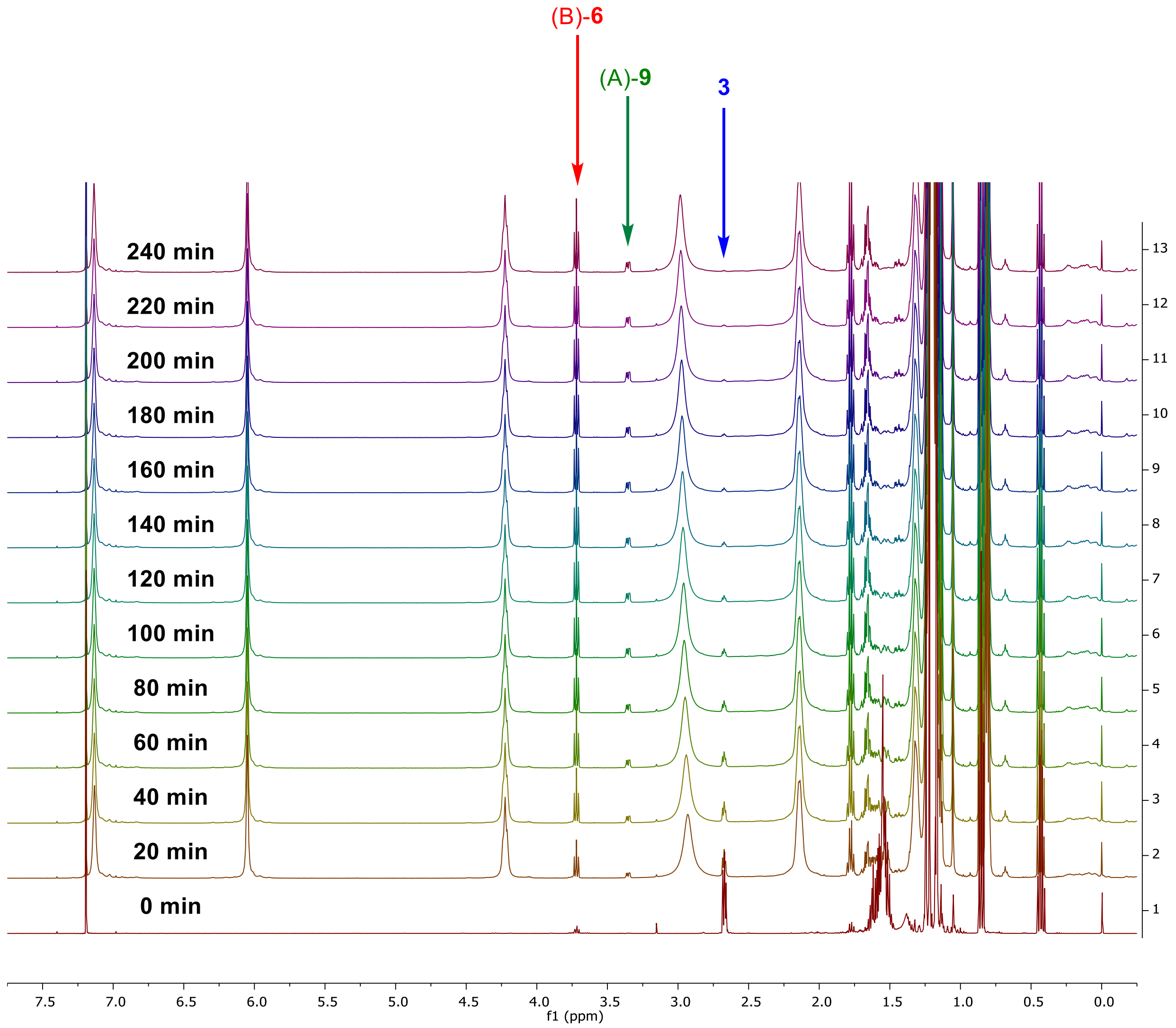

Figure S14. ^{1}H NMR spectra of reaction kinetics at room temperature for the conversion of **3** into (B)-**6** and (A)-**9** in the presence of capsule **15** in $CDCl_3$.

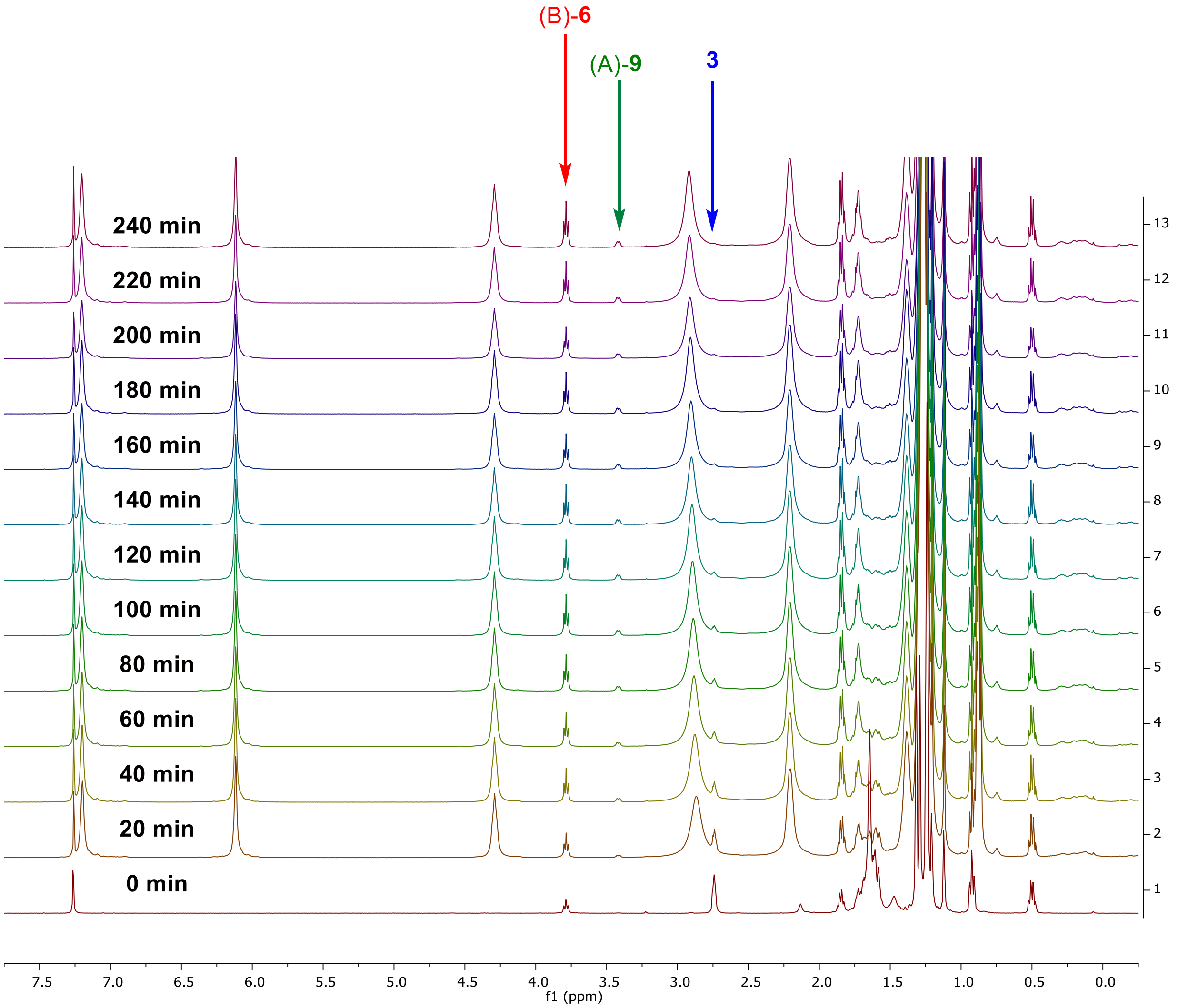

Figure S15. ^{1}H NMR spectra of reaction kinetics at room temperature for the conversion of **3** into (B)-**6** and (A)-**9** in the presence of capsule **15** and 0.25 equivalents of **6** added at the beginning of the reaction in $CDCl_3$.

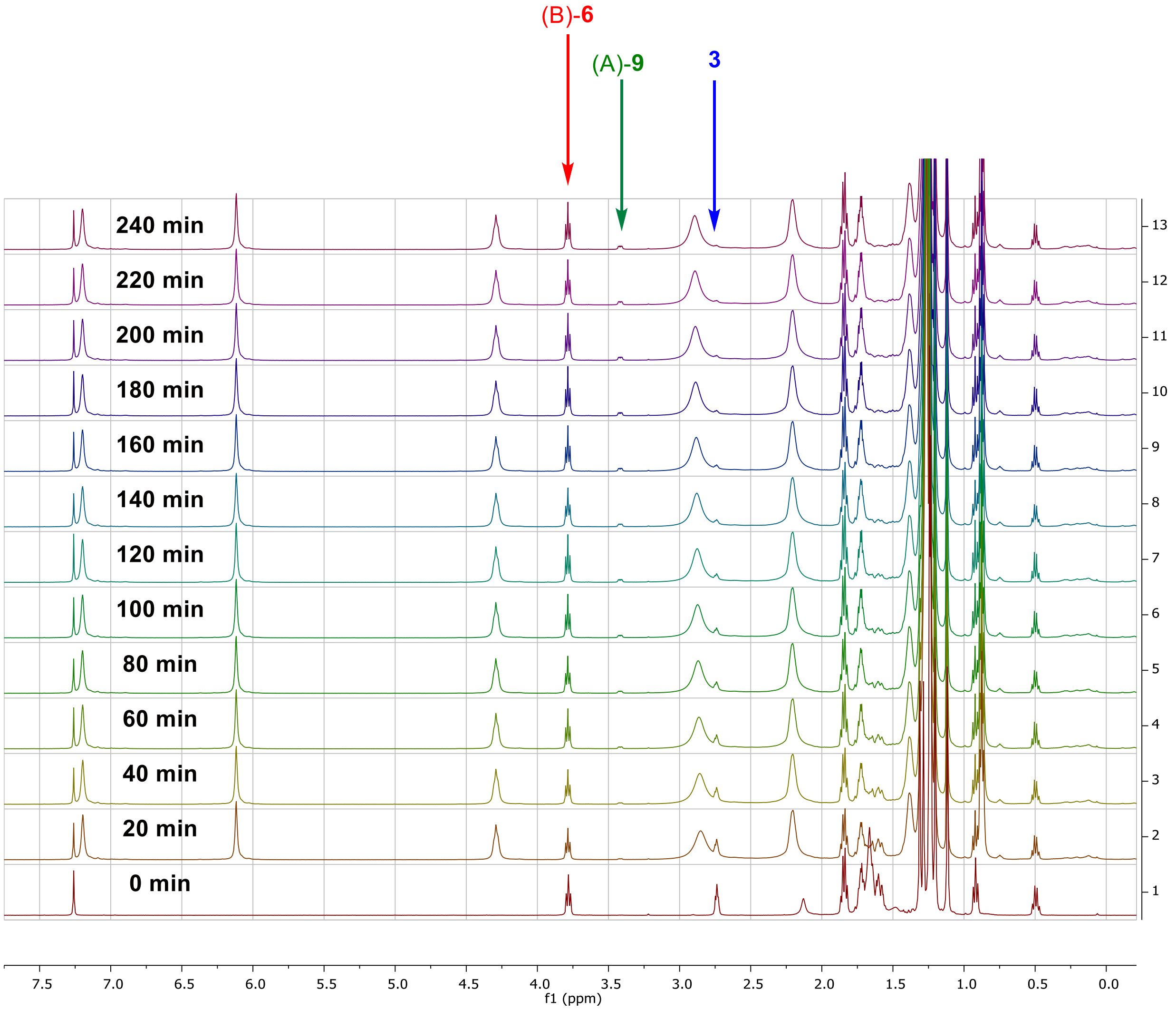

Figure S16. ^{1}H NMR spectra of reaction kinetics at room temperature for the conversion of **3** into (B)-**6** and (A)-**9** in the presence of capsule **15** and 1.0 equivalents of **6** added at the beginning of the reaction in $CDCl_3$.

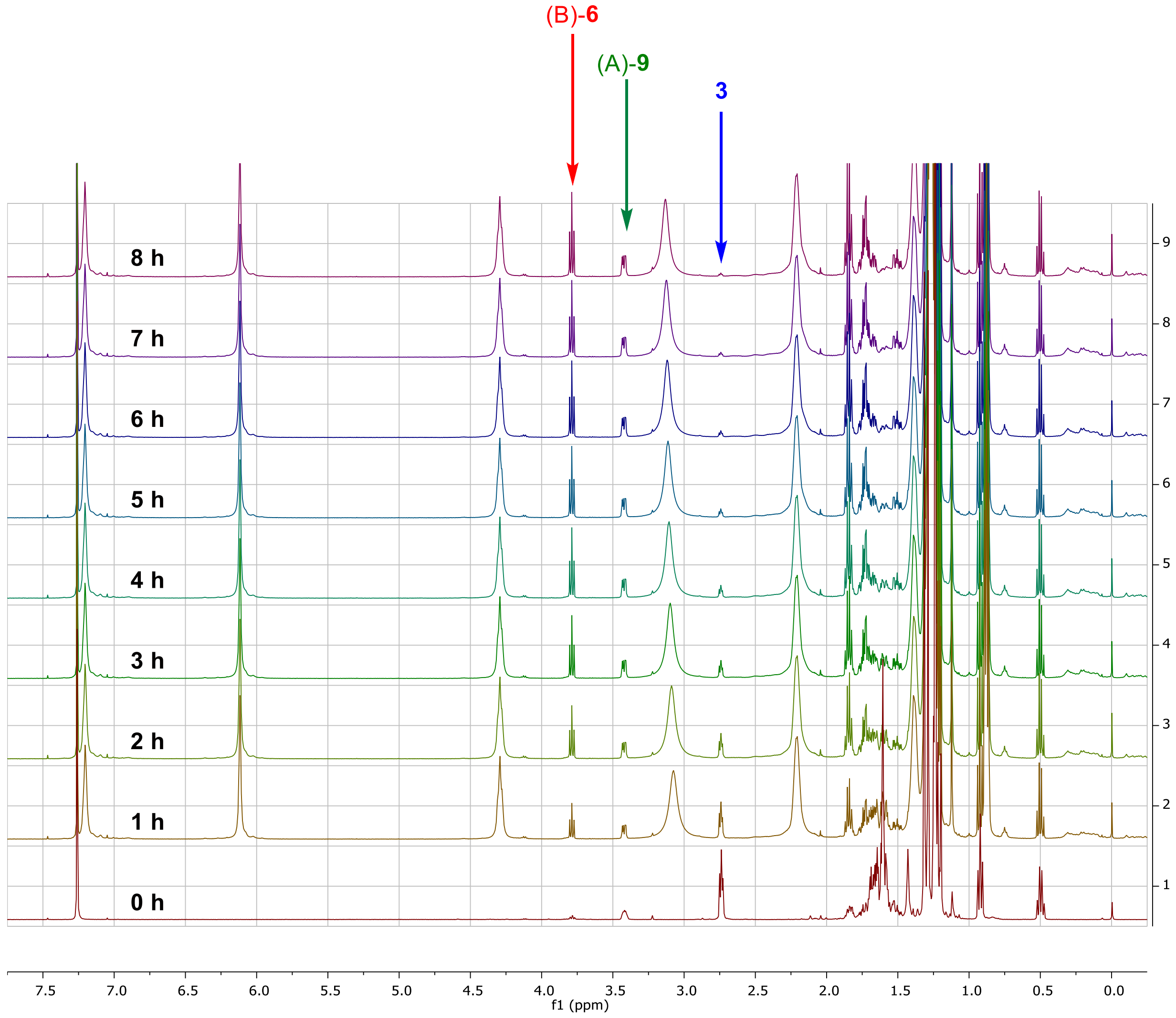

Figure S17. ^{1}H NMR spectra of reaction kinetics at room temperature for the conversion of **3** into (B)-**6** and (A)-**9** in the presence of capsule **15** and 0.25 equivalents of **9** added at the beginning of the reaction in $CDCl_3$.

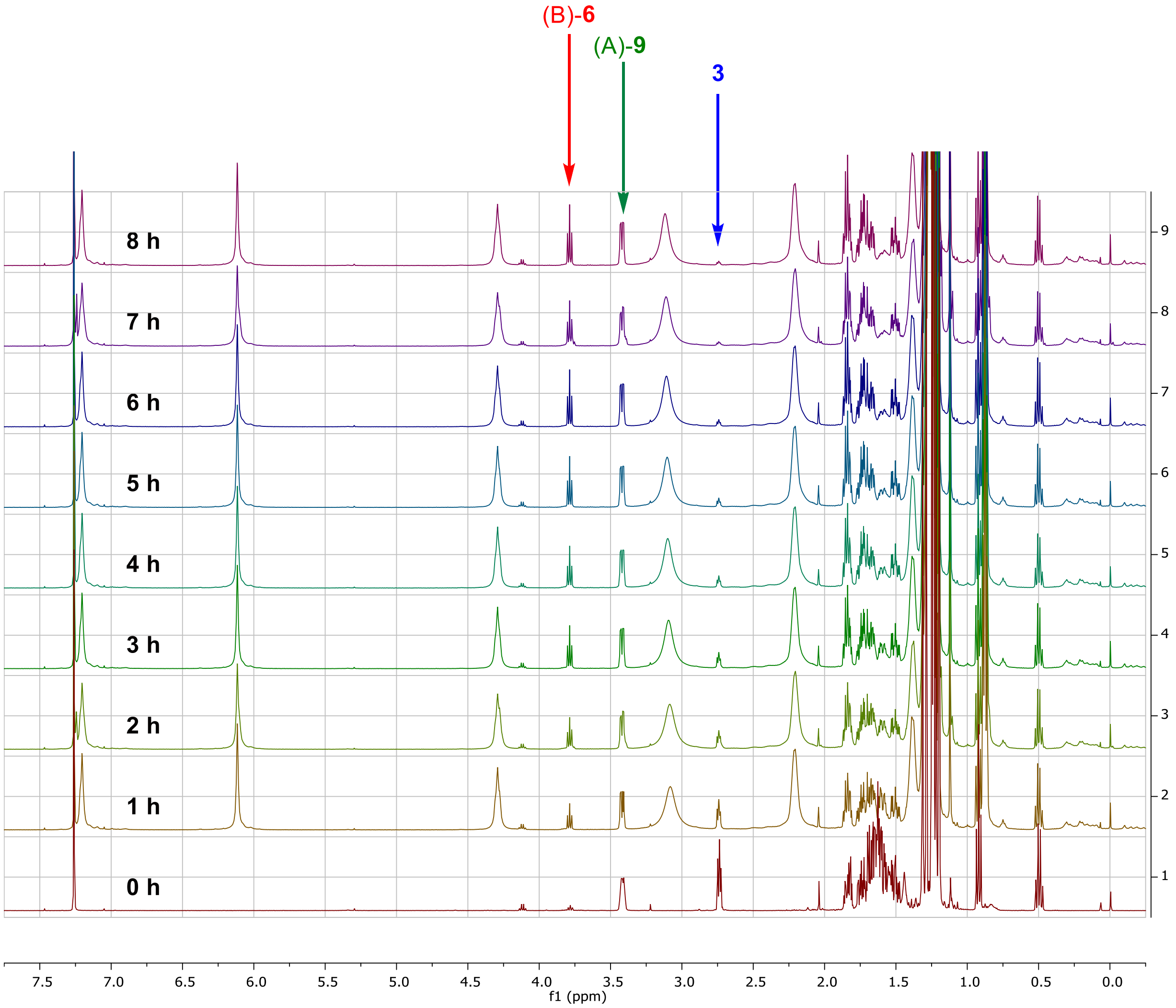

Figure S18. ^{1}H NMR spectra of reaction kinetics at room temperature for the conversion of **3** into (B)-**6** and (A)-**9** in the presence of capsule **15** and 1.0 equivalents of **9** added at the beginning of the reaction in $CDCl_3$.

4. Catalysis

4.1. Co-Catalyst Screening

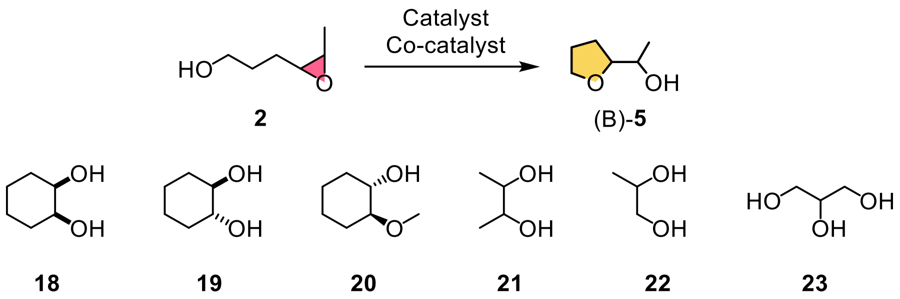

Scheme S3. Product mimics **18-23** tested as co-catalysts.

General procedure C: Solutions of substrate **2** (840 mM, Note: substrate concentration was changed to 460 mM when using **18**, **19** and **20** as the additives), internal standard dibromomethane (1.0 equiv), anion-π catalyst **11** (10 mol%) and additive (1.0 equiv) were mixed in CD_2Cl_2, and stirred at 20 °C for 5 days. The substrate conversion was obtained from the ^{1}H NMR analysis of crude reaction mixture.

4.2. Catalyst Comparison on the Mono-Epoxide Level

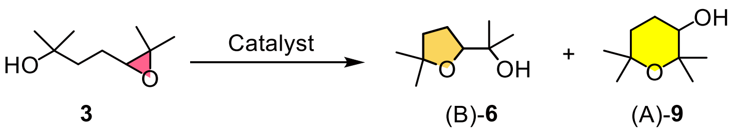

Scheme S4. Chemoselectivity options available for catalyst comparison with mono-epoxide **3**.

Catalysis with AcOH: To a solution of **3** (2.1 M) and CH_2Br_2 (0.3 M) in CD_2Cl_2 was added AcOH (100 mol%), then the mixture was stirred at 40 °C for 18 h. The conversion was calculated from the ^{1}H NMR spectrum of the reaction mixture by comparing the integrals of the signals assigned

to the substrate and the internal standard (CH_2Br_2). The starting material was converted only into Baldwin and anti-Baldwin cyclic ethers.

Catalysis with $SbCl_3$: To a solution of **3** (1.0 M) in CD_2Cl_2 was added $SbCl_3$ (1 mol%), then the mixture was stirred at rt. The starting material was completely consumed after < 5 minutes. The reaction mixture was diluted with CH_2Cl_2, washed twice with 1 M NaOH aqueous solution, once with water, dried over Na_2SO_4 and the solvent removed under reduced pressure. The conversion was calculated from the ^{1}H NMR spectrum of the reaction mixture by comparing the integrals of the signals assigned to the substrate and the products. The starting material was converted only into Baldwin and anti-Baldwin cyclic ethers.

Catalysis with BF_3OEt_2: To a solution of **3** (1.0 M) in CD_2Cl_2 was added BF_3OEt_2 (10 mol%), then the mixture was stirred at rt. The starting material was completely consumed after < 5 minutes. The conversion was calculated from the ^{1}H NMR spectrum of the reaction mixture by comparing the integrals of the signals assigned to the substrate and the products. The starting material was converted only into Baldwin and anti-Baldwin cyclic ethers.

Catalysis with C_6F_6: **3** (1.0 M) and CH_2Br_2 (140 mM) was mixed in C_6F_6 (0.1 mL), then the mixture was stirred at rt for 9 h. The conversion was calculated from the ^{1}H NMR spectrum of the reaction mixture by comparing the integrals of the signals assigned to the substrate and the internal standard (CH_2Br_2). The starting material was converted only into Baldwin and anti-Baldwin cyclic ethers.

Catalysis with 12: To a solution of **3** (2.1 M) and CH_2Br_2 (0.3 M) in CD_2Cl_2 was added **12** (5 mol%), then the mixture was stirred at rt for 30 h. The conversion was calculated from the ^{1}H NMR spectrum of the reaction mixture by comparing the integrals of the signals assigned to the substrate

and the internal standard (CH_2Br_2). The starting material was converted only into Baldwin and anti-Baldwin cyclic ethers.

Catalysis with 17: To a solution of **3** (1.0 M) and CH_2Br_2 (0.3 M) in CD_2Cl_2 was added **17** (20 mol%), then the mixture was stirred at 40 °C for 6 days. The conversion was calculated from the ^{1}H NMR spectrum of the reaction mixture by comparing the integrals of the signals assigned to the substrate and the internal standard (CH_2Br_2). The starting material was converted only into Baldwin and anti-Baldwin cyclic ethers.

Catalysis with 15: To a 5 mm NMR tube, substrate **3** (33.3 mM) was dissolved into 500 µL $CDCl_3$ (Pre-treated as described in the Materials and Methods section). 10.0 µL TES stocking solution (Prepared by dissolving 40.0 µL TES into 1.00 mL $CDCl_3$. This stocking solution was stored in -20 ºC freezer and warmed to room temperature before use) was injected as internal standard. After that, compound **15** (10 mol%) was added, then the mixture was stirred at rt for 4 h. The reaction conversion was obtained by ^{1}H NMR analysis.

Catalysis with 13: To a solution of **3** (2.4 M) in CD_2Cl_2 was added **13** (100 mol%), then the mixture was stirred at 40 °C for 24 h. The consumption of the starting material was followed by ^{1}H NMR spectroscopy. The conversion was calculated from the ^{1}H NMR spectrum of the reaction mixture by comparing the integrals of the signals assigned to the substrate and the catalyst (used also as internal standard). The starting material was converted only in Baldwin and anti-Baldwin cyclic ethers.

Catalysis with 14: To a solution of **3** (1.0 M) in CD_2Cl_2 was added **14** (1 mol%), then the mixture was stirred at rt for 5 min. The consumption of the starting material was followed by ^{1}H NMR spectroscopy. The conversion was calculated from the ^{1}H NMR spectrum of the reaction mixture by

comparing the integrals of the signals assigned to the substrate and the products. The starting material was converted only in Baldwin and anti-Baldwin cyclic ethers.

4.3. Catalyst Comparison on the Di-Epoxide Level

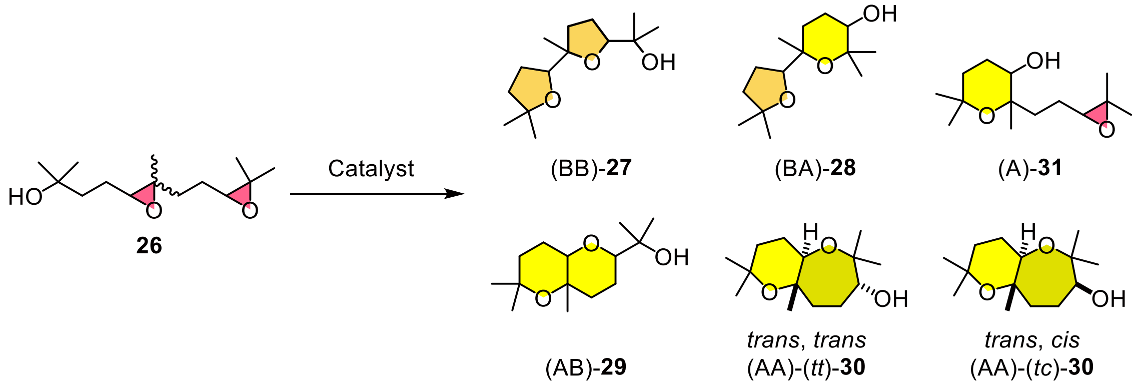

Scheme S5. Chemoselectivity options available for catalyst comparison with di-epoxide **26**.

Catalysis with AcOH: To a solution of **26** (2.1 M) and CH_2Br_2 (0.3 M) in CD_2Cl_2 was added AcOH (100 mol%), then the mixture was stirred at 40 °C for 18 h. The conversion was calculated from the ^{1}H NMR spectrum of the reaction mixture by comparing the integrals of the signals assigned to the epoxides and the internal standard (CH_2Br_2). The starting material was converted only in Baldwin poly-cyclic ethers.

Catalysis with $SbCl_3$: To a solution of **26** (1.0 M) in CD_2Cl_2 was added $SbCl_3$ (100 mol%), then the mixture was stirred at rt for 5 min. The reaction mixture was diluted with $CDCl_3$, washed twice with 1 M NaOH aqueous solution, once with water, and dried over Na_2SO_4. The conversion was calculated from the ^{1}H NMR spectrum of the reaction mixture by comparing the integrals of the signals assigned to the epoxides and the products. The starting material was converted only in Baldwin and anti-Baldwin poly-cyclic ethers.

Catalysis with 12: To a solution of **3** (0.25 M) and CH_2Br_2 (140 mM) in CD_2Cl_2 was added **12** (20 mol%), then the mixture was stirred at rt for 20 h. The conversion was calculated from the ^1H NMR spectrum of the reaction mixture by comparing the integrals of the signals assigned to the substrate and the internal standard (CH_2Br_2). The starting material was converted only into Baldwin and anti-Baldwin poly-cyclic ethers.

Catalysis with 15: To a 5 mm NMR tube, substrate **26** (33.3 mM) was dissolved into 500 µL $CDCl_3$ (Pre-treated as described in the Materials and Methods section). 10.0 µL TES stocking solution (Prepared by dissolving 40.0 µL TES into 1.00 mL $CDCl_3$. This stocking solution was stored in -20 ºC freezer and warmed to room temperature before use) was injected as internal standard. After that, compound **15** (10 mol%) was added, then the mixture was stirred at 30 ºC for 4 days. The reaction conversion was obtained by ^1H NMR analysis.

Catalysis with 13: To a solution of **26** (2.0 M) in CD_2Cl_2 was added **13** (500 mol%), then the mixture was stirred at 60 °C for 2 days. The consumption of the starting material was followed by ^1H NMR spectroscopy. The conversion was calculated from the ^1H NMR spectrum of the reaction mixture by comparing the integrals of the signals assigned to the epoxides and the catalyst (used also as internal standard). The starting material was converted only in Baldwin and anti-Baldwin poly-cyclic ethers.

Catalysis with 14: To a solution of **26** (1.0 M) in CD_2Cl_2 was added **14** (1 mol%), then the mixture was stirred at rt for 2 h. The consumption of the starting material was followed by ^1H NMR spectroscopy. The conversion was calculated from the ^1H NMR spectrum of the reaction mixture by comparing the integrals of the signals assigned to the epoxides and the internal standard (CH_2Br_2). The starting material was converted only in Baldwin and anti-Baldwin polycyclic ethers.

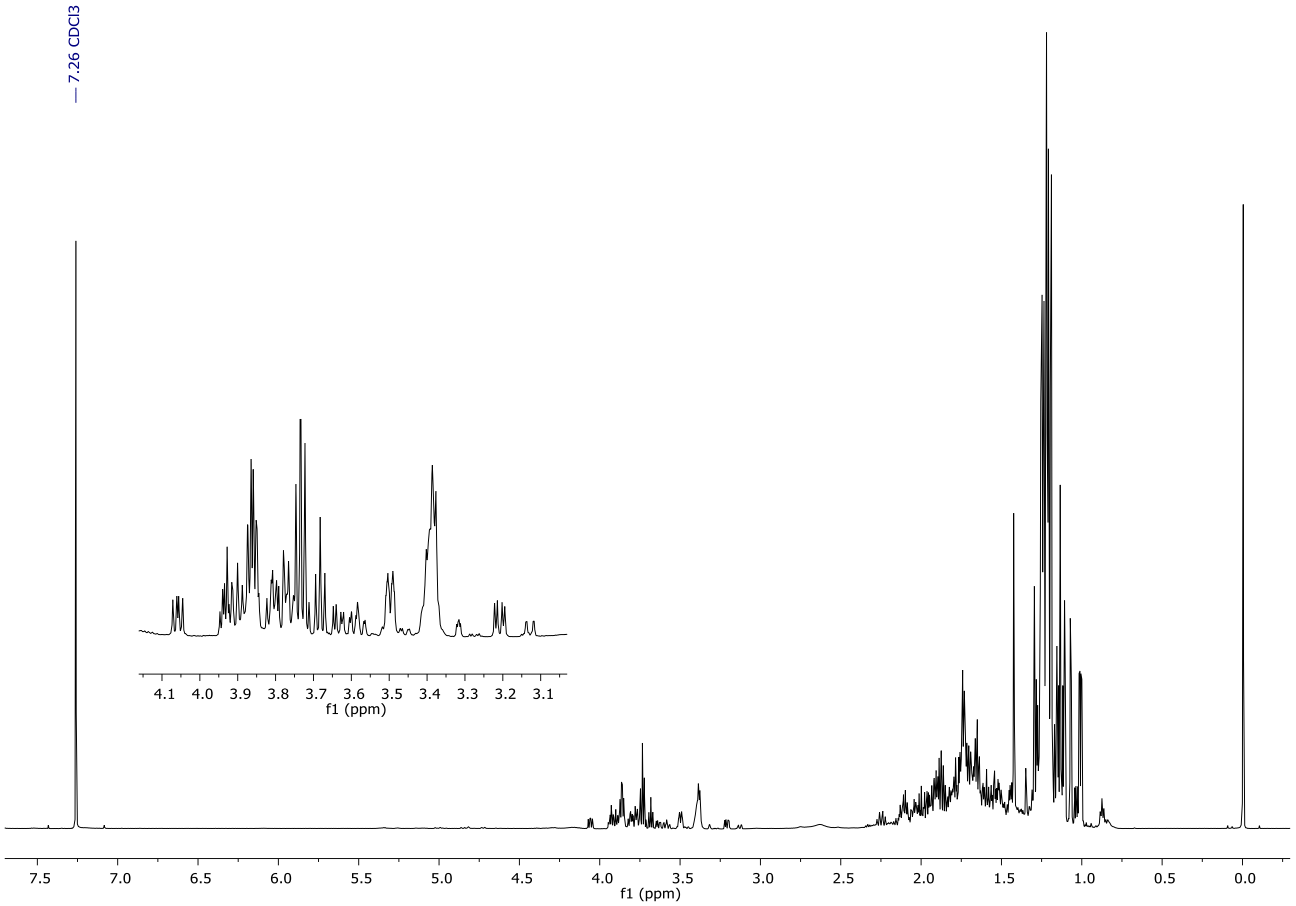

Figure S19. ^{1}H NMR spectrum of the reaction mixtures with substrate **26** and capsule catalyst **15** in $CDCl_3$ (The zoomed spectrum was analyzed in details in manuscript, Figure 7).

4.4. Asymmetric Autocatalysis Assay

General procedure D: To a solution of (*rac*)-cis-**2** (1 M), CH_2Br_2 (140 mM) and the corresponding chiral product (50 mol%) in CD_2Cl_2 was added **12** (10 mol%), then the mixture was stirred at rt. To a vial with Et_2O (0.5 mL), 10 μL reaction mixture was added after indicated time, then the sample was analyzed by chiral GC.

General procedure E: A mixture of (*rac*)**-3** (1 M), CH_2Br_2 (140 mM) and the corresponding chiral product (5 equiv.) was dissolved in **10**, then the solution was stirred at rt. To a vial with Et_2O (0.5 mL), 10 μL reaction mixture was added after indicated time, then the sample was analyzed by chiral GC.

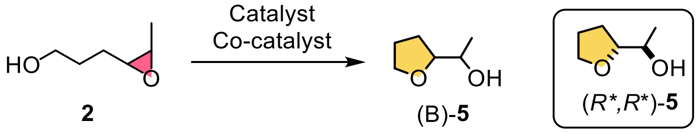

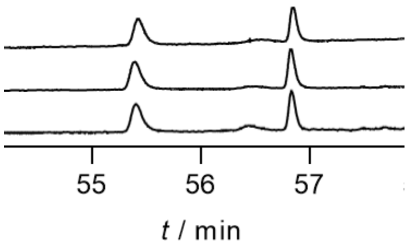

Peak #	RetTime [min]	Type	Width [min]	Area [pA*s]	Height [pA]	Area %
1	55.487	MM	0.1093	7.93552	1.20998	53.17674
2	56.886	MM	0.0750	6.98740	1.55277	46.82326
1	55.409	MM	0.1180	23.38531	3.30404	50.37550
2	56.842	MM	0.0806	23.03668	4.76605	49.62450
1	55.412	MM	0.1181	19.26498	2.71842	50.36237
2	56.839	MM	0.0779	18.98775	4.06082	49.63763

Figure S20. Chiral GC profiles for the consumption of enantiomers of substrate **2** after 25 h (6% ee), 45 h (1% ee**)** and 73 h (1% ee**)** (top to bottom) in the presence of anion-π catalyst **12** and 0.5 equivalents (*R**,*R**)-**5** (86% ee).

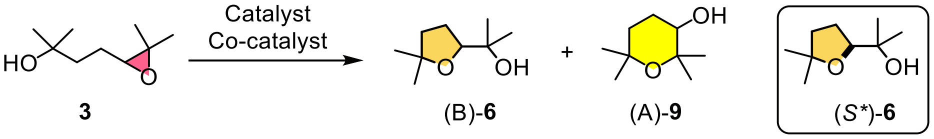

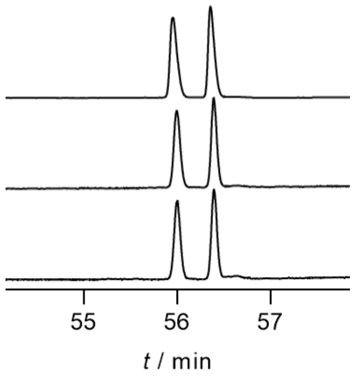

Peak #	RetTime [min]	Type	Width [min]	Area [pA*s]	Height [pA]	Area %
1	55.955	MM	0.0892	143.07100	26.71934	50.08314
2	56.362	MM	0.0790	142.59601	30.06764	49.91686
1	56.002	MM	0.0809	41.07037	8.45628	49.76999
2	56.396	MM	0.0700	41.44997	9.87303	50.23001
1	56.008	MM	0.0796	26.66339	5.58421	50.05205
2	56.398	MM	0.0698	26.60793	6.35299	49.94795

Figure S21. Chiral GC profiles for the consumption of enantiomers of substrate **3** after 2 h (0% ee), 3 h (0 % ee) and 5 h (0% ee) (top to bottom) in the presence of anion-π catalyst **10** (solvent) and 5 equivalents of (*S**)-**6** (79% ee).

5. NMR Spectra

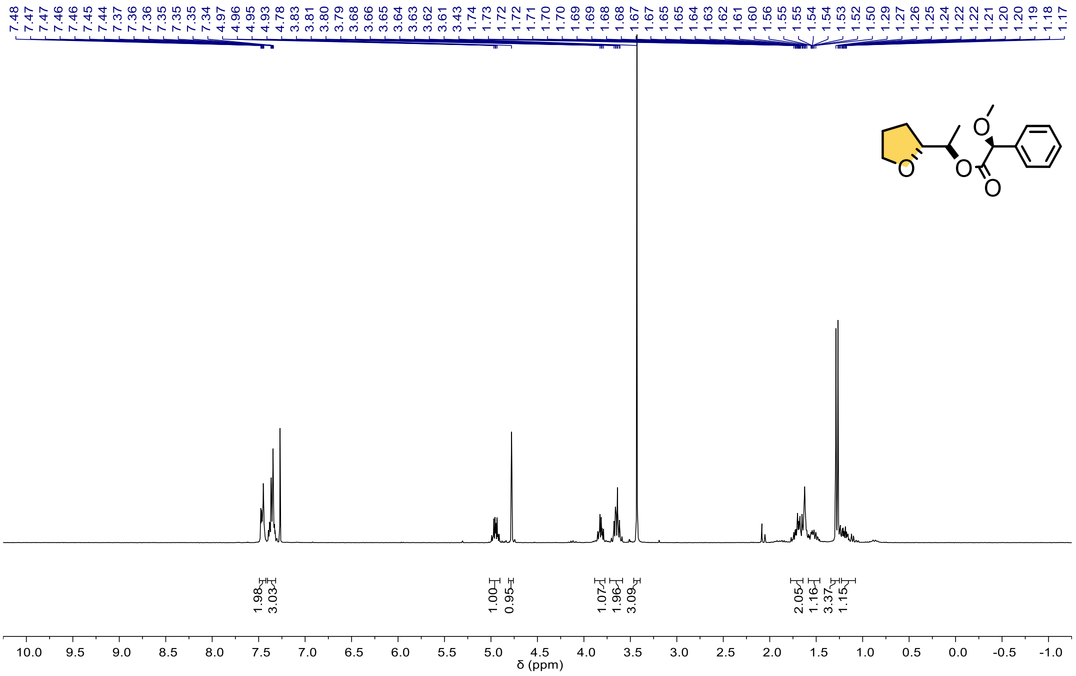

Figure S22. 300 MHz ^{1}H NMR spectrum of (*R**,*R**,*S*)-**25** in $CDCl_3$.

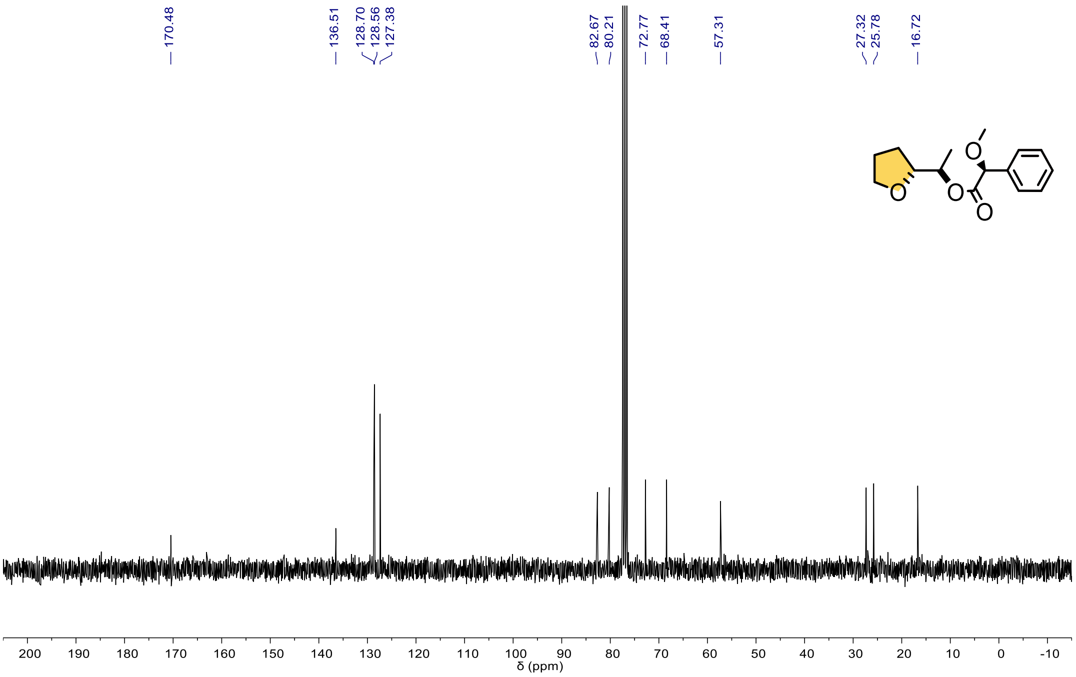

Figure S23. 75 MHz ^{13}C NMR spectrum of (*R**,*R**,*S*)-**25** in $CDCl_3$.

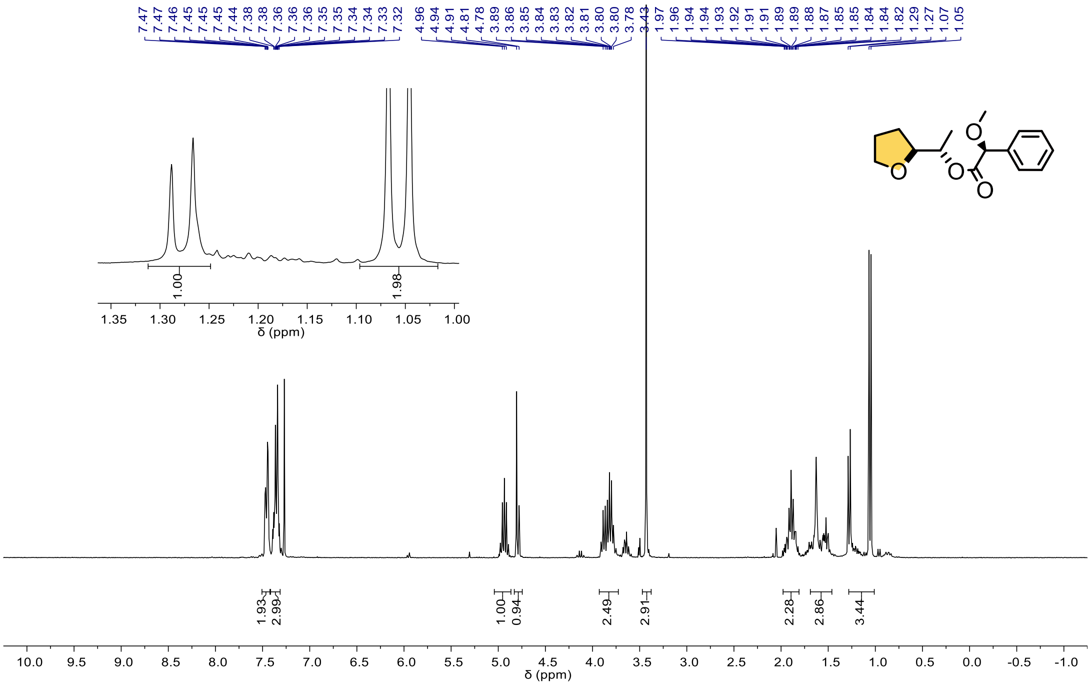

Figure S24. 300 MHz ^{1}H NMR spectrum of (*S**,*S**,*S*)-**25** in $CDCl_3$.

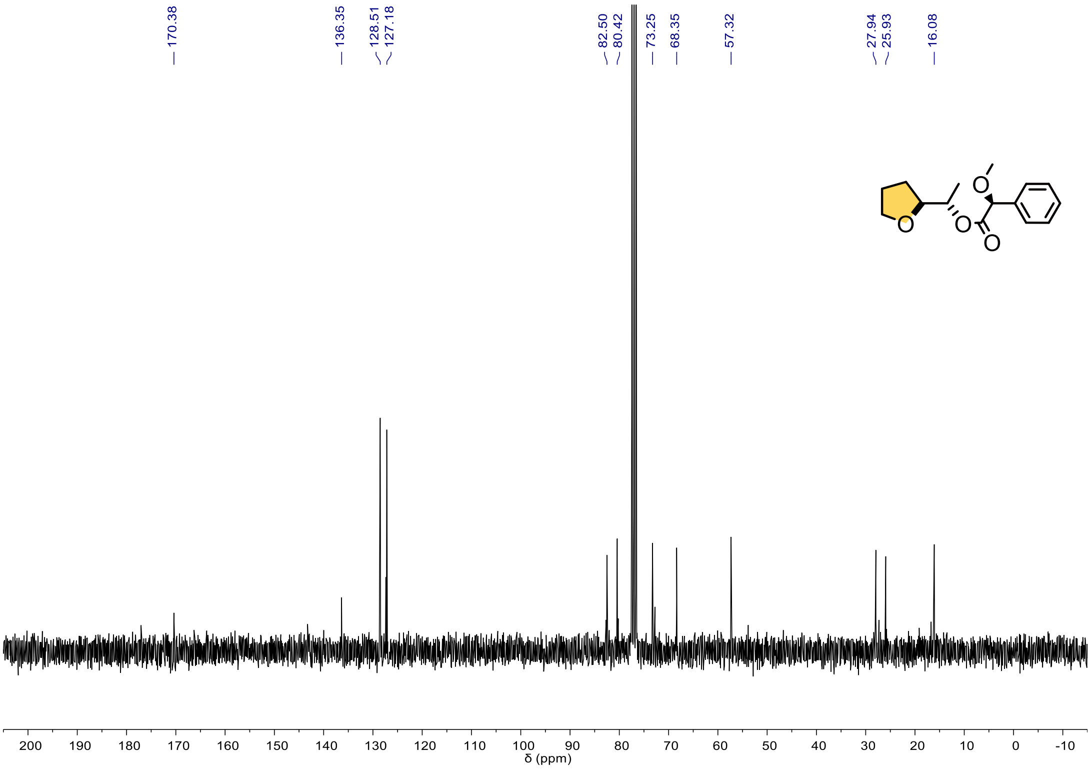

Figure S25. 75 MHz ^{1}H NMR spectrum of (*S**,*S**,*S*)-**25** in $CDCl_3$.

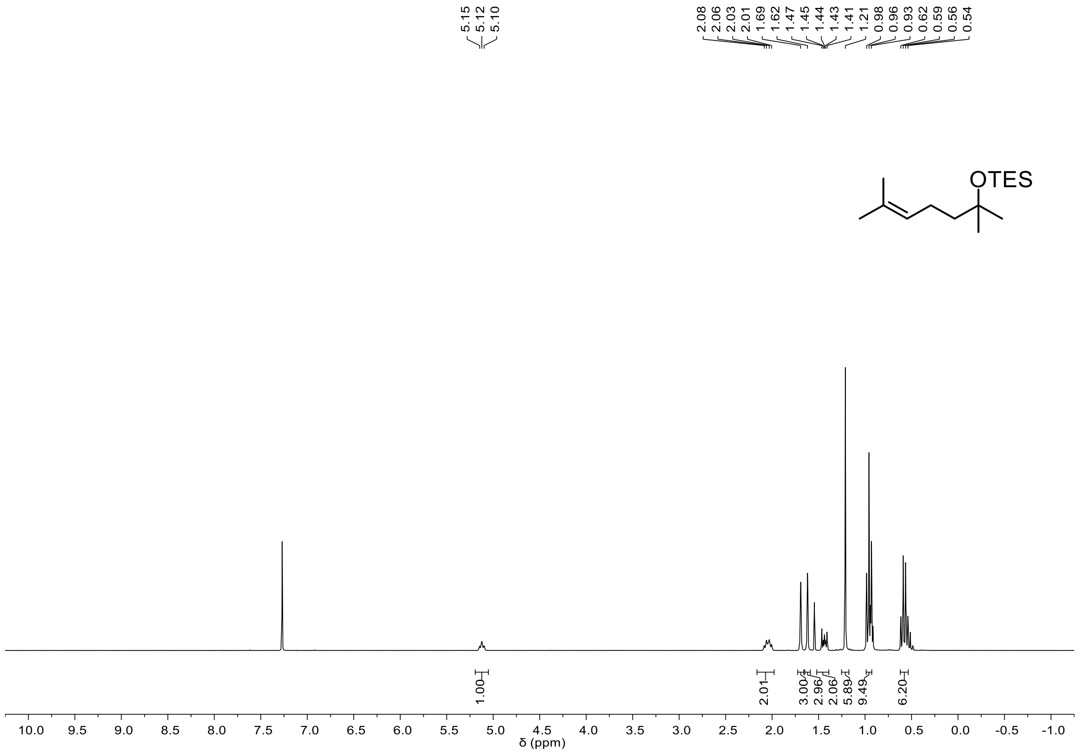

Figure S26. 300 MHz ^{1}H NMR spectrum of **34** in $CDCl_3$.

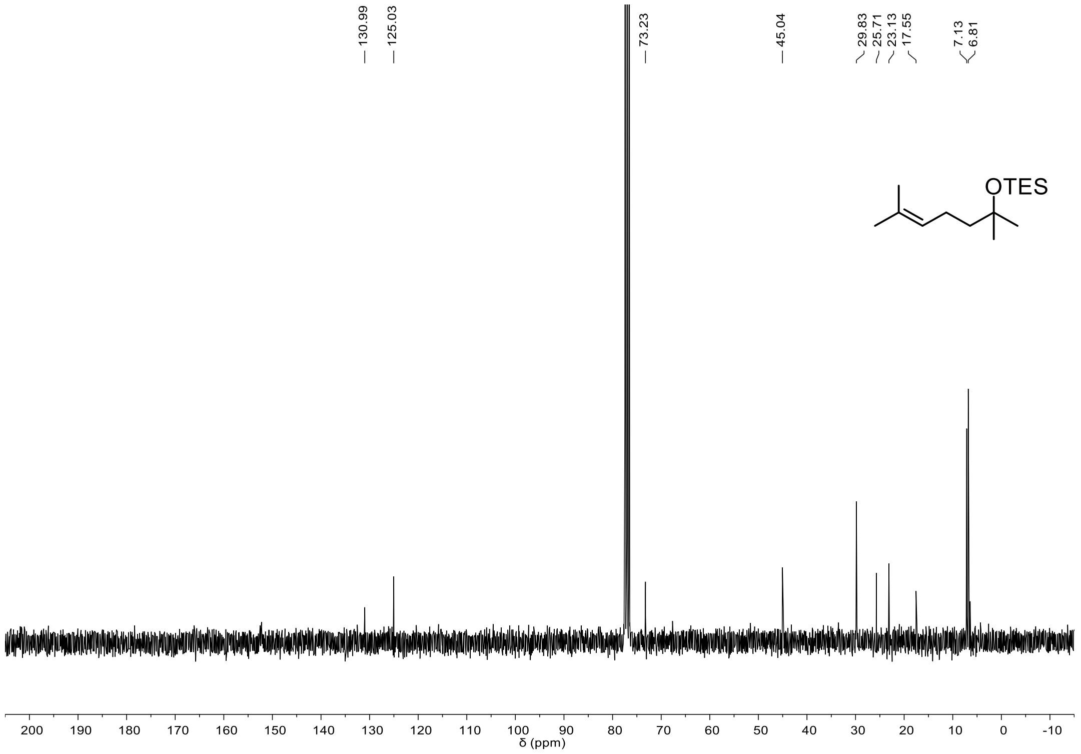

Figure S27. 75 MHz ^{1}H NMR spectrum of **34** in $CDCl_3$.

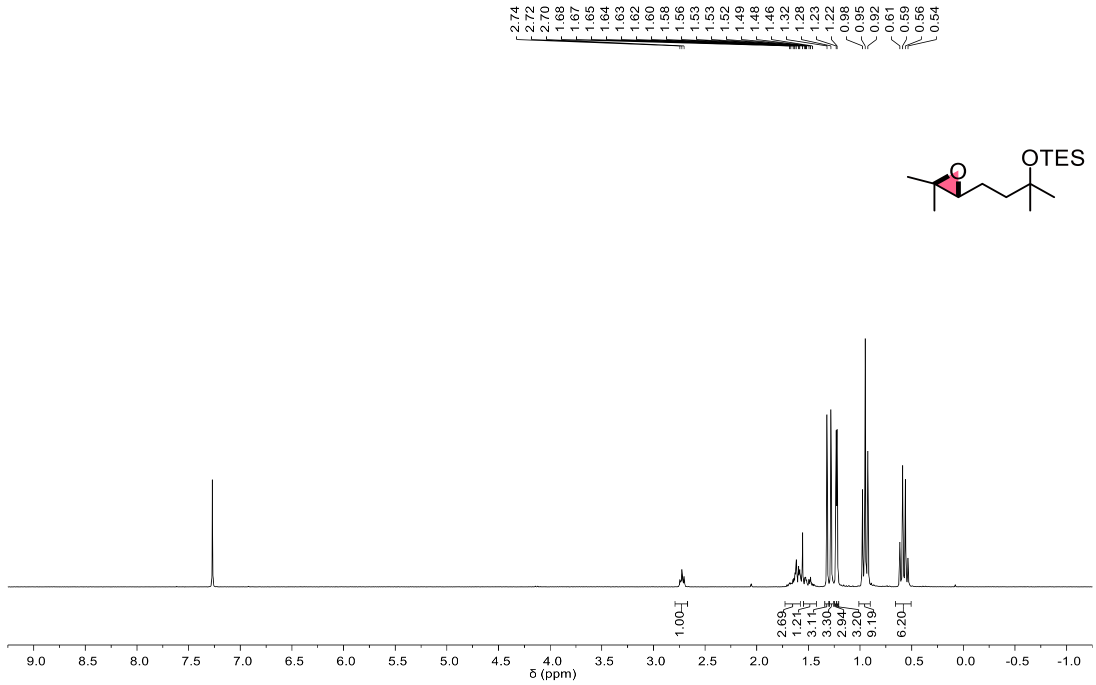

Figure S28. 300 MHz ^{1}H NMR spectrum of **35** in $CDCl_3$.

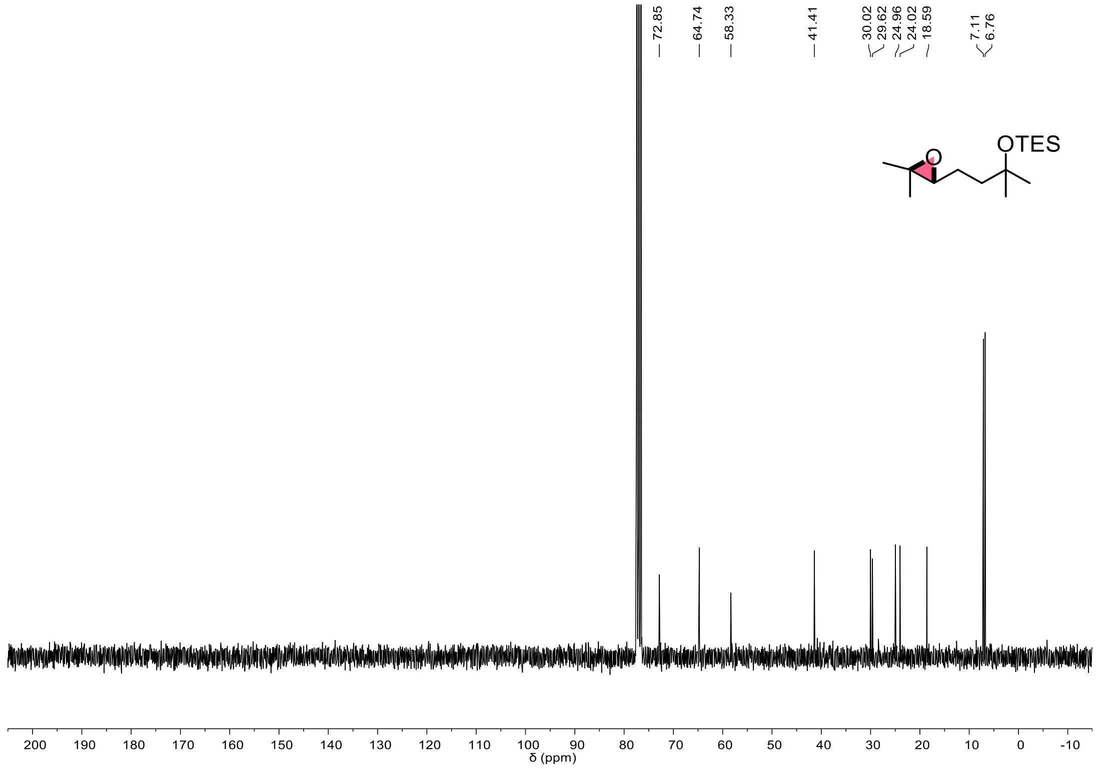

Figure S29. 75 MHz ^{1}H NMR spectrum of **35** in $CDCl_3$.

6. Supporting References

[S1] M. Paraja, X. Hao, S. Matile, *Angew. Chem. Int. Ed.* **2020**, *59*, 15093–15097.

[S2] R. W. Heidebrecht, S. Svenson, C. Zhong, J. A. Gibbs, WO 2017/100744 Al, **2017**.

[S3] J. Lindborg, A. Tanskanen, L. T. Kanerva, *Biocatal. Biotransformation* **2009**, *27*, 204–210.

[S4] S. R. Shenoy, F. R. Pinacho Crisóstomo, T. Iwasawa, J. Rebek, *J. Am. Chem. Soc.* **2008**, *130*, 5658–5659.

[S5] A. Gini, M. Paraja, B. Galmés, C. Besnard, A. I. Poblador-Bahamonde, N. Sakai, A. Frontera, S. Matile, *Chem. Sci.* **2020**, *11*, 7086–7091.

[S6] L. Le, S. Matile, *Supramol. Chem.* **2017**, *29*, 702–706.

[S7] X. Zhang, X. Hao, L. Le, A.-T. Pham, J. López-Andarias, A. Frontera, N. Sakai, S. Matile, *J. Am. Chem. Soc.* **2018**, *140*, 17867–17871.

[S8] J. M. Köster, K. Tiefenbacher, *ChemCatChem.* **2018**, *10*, 2941–2944.

[S9] V. Kozel, C.-G. Daniliuc, P. Kirsch, G. Haufe, *Angew. Chem. Int. Ed.* **2017**, *56*, 15456–15460.

[S10] B. M. Nestl, C. Geinitz, S. Popa, S. Rizek, R. J. Haselbeck, R. Stephen, M. A. Noble, M.-P. Fischer, E. C. Ralph, H. T. Hau, *Nat. Chem. Biol.* **2017**, *13*, 275–281.

[S11] F.-X. Li, S.-J. Ren, P.-F. Li, P. Yang, J. Qu, *Angew. Chem. Int. Ed.* **2020**, *59*, 18473–18478.

[S12] M.-P. Fernando, P.-B. Joaquin F., *J. Chem. Educ.* **1987**, *64*, 925–927.

[S13] L. M. Schwartz, *J. Chem. Educ.* **1989**, *66*, 677.

The original data can be found at: https://doi.org/10.5281/zenodo.4781168
